# Inflammation in Asthma: Mechanistic Insights and the Role of Biologics in Therapeutic Frontiers

**DOI:** 10.3390/biomedicines13061342

**Published:** 2025-05-30

**Authors:** Mohammad Irshad Reza, Nilesh S. Ambhore

**Affiliations:** 1 Department of Pharmaceutical Sciences, North Dakota State University, Fargo, ND 58105, USA; 2 Department of Pharmaceutics, College of Pharmacy, University of Minnesota, Minneapolis, MN 55455, USA

**Keywords:** asthma, biologics, biological therapy, monoclonal antibody, type 1 inflammation, type 2 inflammation

## Abstract

Asthma is a chronic and multifaceted respiratory condition that affects over 300 million individuals across the globe. It is characterized by persistent inflammation of the airways, which leads to episodes of wheezing, breathlessness, chest tightness, and coughing. The most prevalent form of asthma is classified as Type 2 or T2-high asthma. In this variant, the immune response is heavily driven by eosinophils, mast cells, and T-helper 2 (Th2) cells. These components release a cascade of cytokines, including interleukin-4 (IL-4), interleukin-5 (IL-5), and interleukin-13 (IL-13). This release promotes several processes: the production of immunoglobulin E (IgE), which is integral to allergic responses; the recruitment of eosinophils—white blood cells that contribute to inflammation and tissue damage. Conversely, non-Type 2 or T2-low asthma is typically associated with a different inflammatory profile characterized by neutrophilic inflammation. This type of asthma is driven by T-helper 1 (Th1) and T-helper 17 (Th17) immune responses, which are often present in older adults, smokers, and those suffering from more severe manifestations of the disease. Among asthmatic patients, approximately 80–85% of cases are classified as T2-high asthma, while only 15–20% are T2-low asthma. Treatment of asthma focuses heavily on controlling inflammation. Inhaled corticosteroids remain the cornerstone therapy for managing T2-high asthma. For more severe or treatment-resistant cases, biologic therapies targeting specific inflammatory pathways, such as anti-IgE (omalizumab), anti-IL-5 (mepolizumab, benralizumab), and anti-IL-4/IL-13 (dupilumab), have shown great promise. For T2-low asthma, macrolide antibiotics like azithromycin and other novel therapies are being explored. This article reviews the safety, efficacy, and indications of the currently approved biologics and discusses potential novel biologics for asthma.

## 1. Introduction

Asthma is a chronic and multifaceted respiratory condition that affects over 300 million individuals across the globe. It is characterized by persistent inflammation of the airways, which leads to episodes of wheezing, breathlessness, chest tightness, and coughing. This inflammation causes reversible obstruction of airflow and increases the sensitivity of the bronchial tubes to various triggers, such as allergens, irritants, and respiratory infections [1,2,3,4]. At the heart of asthma lies chronic inflammation, which is pivotal in shaping the symptoms experienced by patients, the progression of the disease, and their response to treatment. Importantly, inflammation in asthma is not a one-size-fits-all phenomenon; it varies considerably depending on the specific immunological mechanisms at play and the distinct phenotype of the asthma [5].

Phenotypes refer to the observable characteristics or traits of asthma, such as age of onset, symptom severity, and triggers. In contrast, endotypes describe the underlying biological mechanisms, such as Type 2 or non-Type 2 inflammation, that drive these phenotypes [6]. Table 1 summarizes asthma phenotypes, immune endotypes, and treatment responses.

The most prevalent form of asthma is classified as Type 2 or T2-high asthma. In this variant, the immune response is heavily driven by eosinophils, mast cells, and T-helper 2 (Th2) cells. These components release a cascade of cytokines, including interleukin-4 (IL-4), interleukin-5 (IL-5), and interleukin-13 (IL-13). This release promotes several processes: the production of immunoglobulin E (IgE), which is integral to allergic responses; the recruitment of eosinophils—white blood cells that contribute to inflammation and tissue damage—infiltrating the lungs; increased mucus production; leading to airway obstruction; and enhanced bronchial sensitivity; making the airways more prone to hyperreactivity. This type of asthma is frequently associated with allergic triggers, such as pollen and dust mites, and generally responds favorably to inhaled corticosteroids (ICS) [7,8]. Conversely, non-Type 2 or T2-low asthma is typically associated with a different inflammatory profile characterized by neutrophilic inflammation. This type of asthma is driven by T-helper 1 (Th1) and T-helper 17 (Th17) immune responses, which are often present in older adults, smokers, and those suffering from more severe manifestations of the disease. T2-low asthma tends to exhibit a limited response to corticosteroids, presenting unique treatment challenges for healthcare providers [9]. This complex immunopathology gives rise to diverse asthma phenotypes and endotypes, including allergic asthma (early-onset, IgE-mediated); eosinophilic asthma (often steroid-responsive); neutrophilic asthma (frequently steroid-resistant); and paucigranulocytic asthma (minimal inflammation but marked airway hyperreactivity) [10,11]. Notably, these subtypes are not absolute; there is considerable overlap among them, and a patient’s phenotype may shift over time due to environmental influences, treatment response, or disease progression. Understanding these immunological mechanisms is crucial for precision medicine. The development of biologics targeting these inflammatory pathways has revolutionized the treatment of moderate-to-severe asthma, especially in patients who are unresponsive to standard corticosteroid therapy.

Regardless of the specific type of asthma, chronic and uncontrolled airway inflammation can lead to significant structural changes in the respiratory tract, a phenomenon referred to as airway remodeling. This remodeling process includes several alterations: thickening of the airway walls due to smooth muscle hypertrophy, the development of subepithelial fibrosis that stiffens the airway, hyperplasia of goblet cells that increases mucus production, angiogenesis that leads to increased blood vessel formation in the airways, and thickening of the basement membrane beneath the epithelium due to subepithelial fibrosis. These structural changes can contribute to persistent airflow limitations, making it more difficult for patients to breathe and reducing the effectiveness of standard asthma therapies [3,12,13,14,15,16].

The relationship between inflammation and asthma is a dynamic, cyclical one [17]. Environmental triggers such as allergens, pollutants, or respiratory infections activate immune cells in the airway, leading to the release of inflammatory mediators. These mediators cause swelling, mucus hypersecretion, and bronchoconstriction, resulting in the hallmark symptoms of asthma: wheezing, coughing, shortness of breath, and chest tightness. Inflammatory processes often persist even when symptoms are not present, highlighting the importance of long-term anti-inflammatory treatment [18]. Diagnostic tools such as fractional exhaled nitric oxide (FeNO), blood eosinophil counts, and sputum analysis help identify the type and severity of airway inflammation and guide personalized treatment plans [19].

In recent years, biologic therapies have brought a new paradigm to the management of moderate-to-severe asthma, primarily in those patients who remain symptomatic despite optimal use of ICS and other controller medications [20,21]. By selectively targeting the most crucial molecules involved in Type 2 inflammation, like IgE, IL-5, IL-4, and IL-13, biologics offer an even more specific and individualized treatment method that reaches beyond symptom relief to address the underlying immune dysregulation. These treatments have reported substantial improvements, including reduced rates of exacerbations, improved lung function, and quality of life [20,21]. As our understanding of asthma endotypes becomes clearer, biologics are not just redefining current therapy modalities but also leading the way towards novel interventions targeting discrete inflammatory profiles. This article provides a comprehensive review of currently approved biologics, their mechanism of action, clinical efficacy, and the emerging pipeline of future-generation biologics that will further enhance asthma treatment. This review is based on literature identified through searches of PubMed, Scopus, and Google Scholar databases using keywords including “asthma”, “biologics”, “airway inflammation”, “phenotypes”, and “endotypes”. Preference was given to peer-reviewed articles, clinical trials, and high-quality reviews published in English between 2010 and 2025. Articles were selected based on relevance to the mechanisms of asthma inflammation and the clinical application of biologic therapies.

**Table 1:** Asthma phenotypes, immune endotypes, and treatment responses [4,6,22].

Phenotype	Endotype/Immune Pathway	Key Immune Cells/Cytokines	Clinical Features	Treatment Response
Allergic Asthma	Type 2 (T2-high)	Th2, IL-4, IL-5, IL-13, IgE	Early-onset, atopy, seasonal/perennial triggers	Good response to ICS, anti-IgE, anti-IL-4/13
Eosinophilic Asthma	Type 2 (T2-high)	IL-5, eosinophils, IL-13	Often late-onset, severe, frequent exacerbations	Responds to ICS, anti-IL-5
Non-allergic Asthma	Type 1/17 (T2-low)	Th1, Th17, IFN-γ, TNF-α, IL-17	Adult-onset, more severe, resistant to corticosteroids	Biologics under investigation
Neutrophilic Asthma	Type 1/17 (T2-low)	IL-17, IL-6, neutrophils	Persistent symptoms, steroid-resistant	Biologics under investigation
Paucigranulocytic Asthma	Mixed/Undefined	Low inflammation, unclear cytokine profile	Mild symptoms, airway hyperresponsiveness	Limited response to ICS; treatment based on symptoms

## 2. Mechanistic Insights into Asthma Inflammation

### 2.1. Type 1 Inflammation in Asthma

This refers to an immune response dominated by Th1 cells and associated pro-inflammatory cytokines such as interferon-gamma (IFN-γ) and tumor necrosis factor-alpha (TNF-α) [23]. While asthma has traditionally been understood as a Type 2 (Th2)-mediated disease, particularly in allergic asthma, there is increasing recognition that non-Type 2 asthma—which includes Type 1 and Type 17 (Th17) pathways—plays a significant role, especially in severe, adult-onset, and corticosteroid-resistant forms of the disease [4,24]. Type 1 inflammation primarily arises in response to intracellular pathogens, including various viruses and certain bacterial strains. This process begins when Th1 cells are activated, leading them to secrete IFN-γ. This cytokine plays a crucial role in promoting the activation of macrophages, thereby enhancing their ability to engulf and destroy pathogens and fostering a cytotoxic immune response aimed at eliminating these intracellular threats [25,26].

When this inflammatory pathway is involved in asthma, it manifests as neutrophilic airway inflammation. This differs significantly from the more commonly recognized eosinophilic inflammation observed in classic allergic asthma. Neutrophilic asthma is often characterized by its severity and resistance to traditional treatments, especially ICS. This particular phenotype is frequently associated with various environmental exposures, such as tobacco smoke, irritants encountered in specific occupational settings, air pollution, or ongoing chronic infections that exacerbate the inflammatory response [27].

The presence of Th1-driven inflammation within asthma presents a challenge for treatment. Standard therapies, such as corticosteroids and leukotriene modifiers, predominantly target the Type 2 inflammatory pathways associated with eosinophilic asthma. Consequently, individuals exhibiting neutrophilic asthma may necessitate alternative treatment approaches [28]. These may include the use of macrolide antibiotics, which offer not only antibacterial properties but also immunomodulatory effects that can ameliorate inflammation. Additionally, biologic therapies that specifically target inflammatory mediators like interleukin-6 (IL-6) or TNF-α may be beneficial [29]. In some cases, especially where inflammation and airway remodeling are significant, bronchial thermoplasty could be considered as an innovative treatment option to reduce both the frequency and severity of asthma attacks [20,21].

#### 2.1.1. Molecular Pathways of Type 1 Inflammatory Asthma

Type 1 inflammatory asthma represents a subtype of non-Type 2 (T2-low) asthma, primarily characterized by immune responses led by Th1 cells and their associated cytokines [4,23]. This variant of asthma is less prevalent compared to traditional Type 2 asthma and is typically linked with neutrophilic airway inflammation rather than eosinophilic inflammation. Type 1 asthma frequently occurs in cases of adult-onset asthma, severe asthma, or asthma resistant to corticosteroids and may be triggered or worsened by factors such as environmental pollutants, tobacco smoke, and persistent or recurrent respiratory infections [30].

The essence of Type 1 inflammation centers around the activation and differentiation of Th1 cells. Naïve CD4+ T cells transition into Th1 cells when stimulated by antigen-presenting cells like dendritic cells in the presence of interleukin-12 (IL-12) and IFN-γ. The transcription factor T-bet is crucial for this differentiation, steering the Th1 lineage and inhibiting alternative T cell development, such as Th2 [31,32]. After differentiation, Th1 cells produce IFN-γ, which activates the JAK-STAT1 signaling pathway through the IFN-γ receptor (IFNGR) on various immune and structural cells within the airway [33]. This signaling cascade enhances the transcription of genes associated with inflammation, augments antigen presentation via MHC class II, and raises the expression of adhesion molecules like ICAM-1 and VCAM-1 that facilitate the recruitment of additional immune cells to the inflamed area [33].

Another important cytokine in Type 1 inflammatory asthma is TNF-α. This cytokine, generated by both Th1 cells and classically activated M1 macrophages, plays a role in airway inflammation and remodeling [15,34]. TNF-α binds to its receptors (TNFR1 and TNFR2), initiating downstream pathways such as NF-κB and MAPK, which promote the production of further pro-inflammatory cytokines (e.g., IL-6) and chemokines (e.g., IL-8) [35]. These chemokines attract neutrophils, the primary immune cells involved in Type 1 asthma. Once neutrophils are recruited to the airways, they release harmful mediators like reactive oxygen species (ROS), proteases (e.g., neutrophil elastase), and neutrophil extracellular traps (NETs), leading to epithelial damage, excessive mucus secretion, and airway remodeling [36,37].

In addition to Th1 cells and neutrophils, macrophages play a significant role in Type 1 inflammation. Under the influence of IFN-γ and bacterial components like lipopolysaccharide (LPS), macrophages differentiate into the M1 phenotype, which is pro-inflammatory. M1 macrophages produce large amounts of IL-12, TNF-α, IL-6, and ROS, further escalating local inflammation and inducing tissue damage. This contrasts with the M2 phenotype, more common in Type 2 inflammation, which focuses on tissue repair and anti-inflammatory responses [37].

The activation of toll-like receptors (TLRs) on airway epithelial and immune cells is another contributing factor in Type 1 asthma. These receptors detect microbial components (e.g., TLR4 recognizes LPS, TLR3 responds to viral RNA) and activate innate immune responses through NF-κB and MAPK pathways [37]. TLR activation results in heightened production of pro-inflammatory cytokines and chemokines, further increasing neutrophilic infiltration and airway hyperresponsiveness. This reaction to microbial signals helps clarify why respiratory infections often exacerbate symptoms in this subtype of asthma [38,39,40].

Ultimately, the molecular profile of Type 1 inflammatory asthma features a predominance of Th1 cells, increased levels of IFN-γ and TNF-α, and significant recruitment of neutrophils through chemokines such as IL-8 and CXCL1. These mechanisms lead to chronic inflammation, airway damage, and resistance to treatment, especially concerning corticosteroids, which are generally more effective in eosinophilic Type 2 asthma. As we deepen our understanding of Type 1 asthma, new therapeutic strategies, such as TNF-α inhibitors, JAK-STAT pathway blockers, and CXCR2 antagonists, are being explored to reduce neutrophil recruitment [41].

This asthma subtype highlights the diversity within asthma as a condition and emphasizes the necessity for tailored treatment approaches based on the underlying immune mechanisms. Comprehending the molecular pathways involved in Type 1 inflammation is essential to devising effective therapies for patients who do not respond to conventional treatments aimed at Type 2 mechanisms [41].

#### 2.1.2. Role of Key Th1 Cytokines

**Interferon-gamma (IFN-γ):** It plays a multifaceted and contradictory role in the pathophysiology of asthma, particularly affecting the differentiation between type 2-high (T2-high) and type 2-low (T2-low) asthma phenotypes. IFN-γ is an essential Th1 cytokine, mainly produced by Th1 cells, natural killer (NK) cells, and CD8+ cytotoxic T cells, and is traditionally associated with the activation of macrophages, enhancement of antigen presentation, and facilitation of cell-mediated immunity. In the realm of T2-high asthma, characterized by Th2-driven eosinophilic inflammation, IFN-γ has historically been regarded as protective. It can inhibit the differentiation of Th2 cells, reduce the production of cytokines such as IL-4, IL-5, and IL-13, limit IgE class switching in B cells, and decrease eosinophilic recruitment. Initial theories suggested that a heightened Th1 response, indicated by increased levels of IFN-γ, could counteract Th2 dominance and mitigate allergic airway inflammation [4,33,42]. However, this protective perspective does not hold true for all asthma phenotypes. In T2-low asthma, which is frequently non-eosinophilic, neutrophilic, and resistant to corticosteroids, IFN-γ may play a detrimental role. Increased levels of IFN-γ have been detected in the airways of patients with severe asthma, particularly those exhibiting neutrophilic inflammation. In this scenario, IFN-γ contributes to airway pathology by promoting the secretion of neutrophil-attracting chemokines such as CXCL9, CXCL10, and CXCL11, thereby enhancing the recruitment of Th1 cells and neutrophils [4,41]. Additionally, IFN-γ has been associated with resistance to corticosteroids, potentially by disrupting glucocorticoid receptor signaling pathways. It can also lead to dysfunction in epithelial cells, heightened barrier permeability, and hindered repair mechanisms, all of which contribute to airway remodeling and chronic inflammation. Respiratory viral infections, which are strong inducers of IFN-γ, may amplify these effects and exacerbate asthma symptoms [43,44]. Overall, IFN-γ serves as a double-edged sword in asthma. While it might exert a regulatory effect in T2-high eosinophilic asthma by suppressing Th2 responses, it adopts a more pro-inflammatory and harmful role in T2-low, neutrophilic asthma, particularly in severe, treatment-resistant instances. Its involvement in inflammation, airway damage, and steroid insensitivity underscores its importance in the pathogenesis of difficult-to-treat asthma and implies that modulating IFN-γ or its downstream effects could represent a potential therapeutic approach for certain patient populations [45].

**Tumor necrosis factor-alpha (TNF-α):** It is a cytokine that promotes inflammation and significantly contributes to the pathophysiology of asthma, especially in severe cases and those resistant to corticosteroids. Produced by various immune cells, such as macrophages, T cells, mast cells, and airway epithelial cells, TNF-α plays a role in airway inflammation through several pathways. It is a pleiotropic cytokine that orchestrates multiple pro-inflammatory responses by activating various downstream signaling cascades, including NF-κB, MAPK, and JNK pathways, thereby promoting leukocyte recruitment, airway hyperresponsiveness, and remodeling. It encourages the activation and attraction of neutrophils and eosinophils, increases the contractility of airway smooth muscle, and elevates the expression of adhesion molecules on endothelial cells, which aids in the migration of leukocytes into the airways. TNF-α also participates in airway remodeling by promoting the production of matrix metalloproteinases (MMPs), collagen, and other extracellular matrix components [2,3,15]. In individuals with severe or treatment-resistant asthma, high levels of TNF-α have been detected in the airways, bronchoalveolar lavage fluid, and sputum, which correlates with heightened airway responsiveness, diminished lung function, and ongoing inflammation. Furthermore, TNF-α might hinder the function of glucocorticoid receptors, contributing to resistance to steroids. Although initial studies of anti-TNF treatments (like infliximab and etanercept) indicated some benefits for certain patients with severe asthma, worries about side effects and infection risks have restricted their use in clinical settings. Nonetheless, TNF-α remains an important mediator in non-type 2 (T2-low) asthma and is a potential target for therapy, particularly in cases characterized by neutrophilic inflammation and inadequate response to corticosteroids.

### 2.2. Type 2 Inflammation in Asthma

Type 2 (T2) inflammation plays a crucial role in the pathophysiology of T2-high asthma, which is the most prevalent phenotype. This immune response includes Th2 cells and type 2 innate lymphoid cells (ILC2s) that secrete cytokines such as IL-4, IL-5, and IL-13. These cytokines contribute to essential aspects of asthma, including eosinophilic infiltration of the airways, production of IgE, heightened reactivity, excessive mucus production, and changes in airway structure [36,46]. When allergens or irritants are encountered, airway epithelial cells secrete alarmins like IL-25, IL-33, and thymic stromal lymphopoietin (TSLP), which in turn activate Th2 cells and ILC2s. IL-4 enhances IgE production, IL-5 is vital for the functioning of eosinophils, and IL-13 is responsible for mucus production as well as airway remodeling [47]. Basophils and mast cells are critical effector cells in the immunopathogenesis of asthma, particularly in the context of type 2 inflammation. Mast cells, which are resident in the airway epithelium and smooth muscle, release a range of mediators upon activation, including histamine, prostaglandins, leukotrienes, and cytokines such as IL-4 and IL-13 that contribute to bronchoconstriction, mucus production, and recruitment of other inflammatory cells [48]. Basophils, although less abundant, are potent sources of IL-4 and IL-13 and serve as key amplifiers of Th2 cell differentiation and eosinophilic inflammation [49]. Both cell types express high-affinity IgE receptors (FcεRI) and are central to allergen-induced airway hyperresponsiveness and early-phase reactions.

Clinically, T2-high asthma is marked by early onset, atopic conditions, and a favorable response to ICS. Common features include elevated eosinophil counts, increased fractional exhaled nitric oxide (FeNO), and IgE sensitization to various allergens. In more severe instances, biologic therapies that target components of T2 inflammation may be necessary; these include omalizumab (anti-IgE), mepolizumab, reslizumab, benralizumab, dupilumab, and tezepelumab [34,50]. The diagnosis and management of this condition are informed by biomarkers such as blood eosinophil counts and FeNO, which assist in phenotyping and predicting treatment responses, as confirmed in the ORACLE2 study [51]. Genetic and environmental influences play a role in both the emergence and intensity of T2-high asthma. A deeper understanding of T2 inflammation has facilitated personalized and effective treatment strategies, significantly enhancing patient outcomes.

#### 2.2.1. Molecular Pathways of Type 2 Inflammatory Asthma

The molecular mechanisms involved in type 2 (T2) inflammatory asthma revolve around a well-coordinated immune response that includes a complex network of signals from epithelial cells, activation of immune cells, and inflammation mediated by cytokines. This pathway is prevalent in the T2-high asthma phenotype, which is commonly linked with eosinophilic inflammation, IgE-mediated allergies, and responsiveness to corticosteroids. It is influenced by both adaptive immunity (Th2 cells) and innate immunity (type 2 innate lymphoid cells, or ILC2s) and is triggered by environmental factors such as allergens, respiratory viruses, pollutants, and microbes [52].

**Epithelial Activation and Release of Alarmins:** The process of type 2 inflammation in asthma begins at the airway epithelium, which reacts to environmental factors like allergens, pollutants, and viruses. When stimulated, epithelial cells secrete “alarmins”, which include thymic stromal lymphopoietin (TSLP), interleukin-25 (IL-25), and interleukin-33 (IL-33). These alarmins serve as primary mediators that activate both dendritic cells and innate lymphoid cells, initiating the inflammatory cascade [46,53]. Alarmins are also implicated in the activation of basophils and amplification of eosinophilic inflammation, making them attractive therapeutic targets.

**Dendritic Cell Activation and Th2 Cell Differentiation**: Dendritic cells activated by TSLP capture inhaled allergens and move to the lymph nodes, where they present these antigens to naïve CD4+ T cells. Through the expression of OX40 ligand (OX40L), they facilitate the differentiation of these naïve T cells into Th2 cells. Th2 cells subsequently produce the characteristic cytokines of type 2 inflammation—IL-4; IL-5; and IL-13—which coordinate the allergic and eosinophilic responses in asthma [54,55].

**Role of Th2 Cytokines**: The Th2 cytokines perform specific and essential functions in the pathophysiology of asthma. IL-4 assists in class switching for B cells to generate IgE while also promoting the further differentiation of Th2 cells. IL-5 is crucial for the development, recruitment, and activation of eosinophils. IL-13 plays a role in mucus hypersecretion, goblet cell metaplasia, increased airway responsiveness, and tissue remodeling, making it a significant factor in chronic disease progression [53,54,56].

**Innate Lymphoid Cell Type 2 (ILC2) Activation:** ILC2s are directly activated by epithelial alarmins (IL-25, IL-33, and TSLP) without requiring antigen presentation. Once activated, they quickly secrete large amounts of IL-5 and IL-13, contributing to eosinophilic inflammation and changes in airway structure. ILC2s are particularly important in non-allergic eosinophilic asthma and help maintain inflammation even without adaptive immune activation [55,57].

**Effector Phase**: In the effector phase, there is a recruitment of eosinophils, production of IgE, and activation of mast cells. Eosinophils, drawn by IL-5 and chemokines such as eotaxin, release cytotoxic granules that damage the airway tissues. IL-4 and IL-13 stimulate B cells to create IgE, which binds to FcεRI receptors on mast cells and basophils. Upon subsequent exposure to allergens, cross-linking of IgE leads to mast cell degranulation, releasing histamine and other mediators that result in bronchoconstriction and inflammation [55,58].

**Tissue Remodeling and Chronic Inflammation**: Prolonged exposure to type 2 cytokines, particularly IL-13, causes airway remodeling, which is characterized by goblet cell hyperplasia, subepithelial fibrosis, smooth muscle hypertrophy, and thickening of the basement membrane beneath the epithelium due to subepithelial fibrosis and neoangiogenesis, which contributes to increased vascularity and airway inflammation. Eosinophils and mast cells further exacerbate this by releasing enzymes, growth factors, and reactive oxygen species, leading to ongoing structural changes in the airway and a progressive decline in lung function over time [2,46,53].

**Key Molecular Targets and Therapeutics**: The understanding of the molecular pathways involved in type 2 inflammation has led to the creation of targeted biologic treatments. These include omalizumab (anti-IgE), mepolizumab and reslizumab (anti-IL-5), benralizumab (anti-IL-5 receptor α), dupilumab (anti-IL-4 receptor α, which blocks IL-4 and IL-13), and tezepelumab (anti-TSLP). These biologics assist in decreasing exacerbations and enhancing control in patients with moderate to severe T2-high asthma who do not respond well to traditional therapies [54,59].

#### 2.2.2. Role of Key Th2 Cytokines

**Interleukin-4 (IL-4):** IL-4 is crucial for initiating and maintaining the type 2 immune response in asthma. It is mainly produced by activated Th2 cells and is vital for transforming naïve CD4+ T cells into Th2 cells, thereby enhancing the type 2 inflammatory response [60]. IL-4 also facilitates class switching in B cells, resulting in the production of IgE. IgE attaches to high-affinity receptors (FcεRI) on mast cells and basophils, sensitizing them to allergens [60]. When re-exposed to the allergen, cross-linking of IgE triggers the release of histamine and various mediators that induce bronchoconstriction, increase vascular permeability, and enhance mucus production—key characteristics of allergic asthma [61].

**Interleukin-5 (IL-5):** This cytokine is crucial for the development, activation, recruitment, and survival of eosinophils, which are a defining feature of type 2-high asthma [62]. Primarily produced by Th2 cells and type 2 innate lymphoid cells (ILC2s), IL-5 stimulates eosinophil production in the bone marrow and aids in their migration to the airway tissues. Upon arrival in the airways, eosinophils release cytotoxic granules, such as major basic protein and eosinophil peroxidase, which harm epithelial cells, promote airway hyperresponsiveness, and contribute to chronic inflammation and tissue remodeling [63,64]. Higher levels of IL-5 and eosinophils are typically linked with more severe asthma and frequent exacerbations [64].

**Interleukin-13 (IL-13):** This cytokine has a complex role in asthma and is especially associated with airway remodeling and mucus overproduction. Similar to IL-4, it is produced by Th2 cells and ILC2s [65]. IL-13 promotes the proliferation of goblet cells and boosts mucin production, leading to an accumulation of mucus in the airways [66]. It also increases airway hyperresponsiveness (AHR) by influencing smooth muscle contractility and contributes to subepithelial fibrosis and collagen buildup, which result in airway narrowing and persistent airflow obstruction over time [67]. Additionally, IL-13 can enhance the expression of nitric oxide synthase in airway epithelial cells, leading to elevated levels of exhaled nitric oxide (FeNO), a marker often utilized to identify T2-high asthma [68]. IL-4 and IL-13 share one receptor, called type II receptor. This receptor is a heterodimer consisting of an IL-4 receptor (IL-4R) α and an IL-13 receptor (IL-13R) α1 subunit [69].

Together, IL-4, IL-5, and IL-13 coordinate the allergic and eosinophilic inflammation that characterizes type 2 asthma, resulting in symptoms such as wheezing, coughing, excessive mucus production, and bronchial hyperreactivity. These cytokines have emerged as essential therapeutic targets in developing biologic treatments for individuals with moderate to severe asthma [61,64,65].

### 2.3. Type 17 Inflammation in Asthma

The primary drivers of this condition are Th17 cells, known for their production of interleukin-17 (IL-17) and additional pro-inflammatory cytokines such as IL-22 and IL-21 [70]. Although type 2 (T2) inflammation is the main mechanism in numerous asthma cases, type 17 inflammation holds particular significance in non-eosinophilic asthma, particularly in instances that are resistant to steroids and severe [71]. Environmental factors, including airway infections (notably viral), pollutants, and fungal allergens, activate Th17 cells. The signature cytokine of Th17 cells, IL-17, plays a role in neutrophilic inflammation, a characteristic of asthma driven by type 17. It facilitates the recruitment and activation of neutrophils in the airways and boosts the production of pro-inflammatory mediators like IL-8 and granulocyte-macrophage colony-stimulating factor (GM-CSF). This results in airway inflammation, increased responsiveness, and tissue damage [72]. Notably, type 17 inflammation frequently correlates with a poor response to corticosteroids, presenting a significant challenge in managing severe asthma. Gaining insight into the function of Th17 cells and IL-17 in asthma has led to the exploration of targeted therapies, such as IL-17 inhibitors and other immune-modulating treatments, which may offer potential advantages for patients facing difficult-to-treat asthma phenotypes [70].

#### 2.3.1. Molecular Pathways of Type 17 Inflammatory Asthma

The pathophysiology of type 17 inflammatory asthma is intricate, characterized by diverse immune cell interactions and cytokine networks, prominently featuring Th17 cells. These cells arise from naïve CD4+ T cells upon exposure to a milieu of cytokines, including IL-1β, IL-6, and TGF-β, particularly in the presence of IL-23 [73]. The differentiation of Th17 cells is primarily driven by the transcription factor RORγt (retinoic acid receptor-related orphan receptor gamma t), which is crucial for establishing the Th17 phenotype. Following their differentiation, Th17 cells release various cytokines, notably IL-17A, IL-17F, IL-22, and IL-21, with IL-17A being the most extensively studied in the context of asthma pathogenesis [9].

The Th17-mediated inflammatory response in asthma is further augmented by IL-23, a cytokine produced by dendritic cells and macrophages, crucial for Th17 cell longevity and enhancing IL-17 production. IL-23 signaling through its receptor, IL-23R, employs the STAT3 transcription factor, vital for sustaining Th17 responses, thereby nurturing a positive feedback loop where IL-17 amplifies IL-23 synthesis, perpetuating inflammation [9].

A notable aspect of type 17 inflammation in asthma is its inherent steroid resistance. The activation of NF-κB and MAPK pathways due to IL-17 signaling can suppress glucocorticoid receptor (GR) functionality by downregulating histone deacetylase (HDAC) expression, which otherwise promotes glucocorticoid-mediated anti-inflammatory actions [72]. Consequently, patients with IL-17-driven asthma often exhibit a diminished response to corticosteroids, presenting a significant challenge for managing severe asthma cases.

Recent advancements in the molecular understanding of these processes have spurred the development of targeted therapies, including IL-17 inhibitors and other biologic agents. Notables such as secukinumab (an IL-17A monoclonal antibody) and brodalumab (an IL-17 receptor antagonist) have shown potential in clinical trials, particularly in individuals with neutrophilic and corticosteroid-resistant asthma. These therapies are designed to disrupt the IL-17 signaling axis, reducing neutrophil influx, airway inflammation, and improving lung function in patients unresponsive to traditional treatments [9,74].

In brief, type 17 inflammatory asthma is orchestrated by a complex interplay of cytokines, transcription factors, and signaling pathways, with IL-17 serving as a pivotal component of the inflammatory landscape. The activation of Th17 cells and their downstream impacts on neutrophil recruitment, epithelial cell activation, and airway remodeling play crucial roles in the chronic inflammation and persistent airway alterations characteristic of this asthma phenotype [75]. Advancements in deciphering these molecular mechanisms herald new strategies in precision medicine, paving the way for innovative therapeutic options for patients suffering from severe, steroid-resistant asthma.

#### 2.3.2. Role of Key Th17 Cytokines

**IL-17A:** It interacts with its receptor complex, IL-17RA/IL-17RC, present on epithelial cells, fibroblasts, and endothelial cells, among others [76]. This engagement activates a cascade of intracellular signaling pathways, predominant among them the NF-κB (nuclear factor kappa-light-chain-enhancer of activated B cells) and MAPK (mitogen-activated protein kinase) pathways, along with C/EBPβ (CCAAT/enhancer-binding protein beta) [72]. These signaling cascades lead to enhanced pro-inflammatory gene expression, resulting in the secretion of chemokines like CXCL1, CXCL8 (IL-8), and CCL20, which are essential for recruiting neutrophils and monocytes to the airways [42]. Additionally, IL-17 signals also promote the production of prostaglandins and leukotrienes, amplifying the inflammatory response and contributing to bronchoconstriction and AHR [77].

**IL-17F**: This cytokine, closely related to IL-17A, engages the same receptors and exhibits comparable pro-inflammatory effects, although its specific role in asthma is not yet fully elucidated [42]. Recent studies have demonstrated that the expression of IL-17F in the airways is positively associated with both the presence of neutrophils and the severity of asthma [78,79]. Further, the level of IL-17F in the bronchoalveolar lavage fluid (BALF) of mice exposed to toluene diisocyanate (TDI) was found to be higher than in control mice. In alignment with findings from the HDM-induced model, using a monoclonal antibody to neutralize IL-17F significantly reduced neutrophil infiltration in the bronchi [80].

**IL-22:** IL-22, another critical cytokine from Th17 cells, plays a significant role in epithelial barrier dysfunction and mucus hyperproduction, which manifest as hallmark asthma symptoms like wheezing, coughing, and mucus obstruction [81,82]. It signals through the IL-22 receptor (IL-22R1), activating the JAK-STAT3 pathway, leading to increased expression of mucin genes and matrix metalloproteinases (MMPs), thereby facilitating airway remodeling and tissue damage [83].

#### 2.3.3. Biomarkers in Asthma

Asthma is a heterogeneous disease with varied inflammatory pathways. The identification of reliable biomarkers is central to phenotyping asthma, predicting treatment responses, and guiding biologic therapy. Among the most validated biomarkers are blood eosinophil counts, fractional exhaled nitric oxide (FeNO), serum immunoglobulin E (IgE), and periostin levels.

##### Blood Eosinophil Count

Blood eosinophils are a surrogate marker of Type 2 (T2) inflammation and correlate with airway eosinophilia. Elevated eosinophil levels (≥150–300 cells/μL) are predictive of good response to anti-IL-5 therapies (mepolizumab, reslizumab, benralizumab) and are associated with increased exacerbation risk in severe asthma. Eosinophil levels can fluctuate but remain a cost-effective and widely accessible biomarker [84].

##### Fractional Exhaled Nitric Oxide (FeNO)

FeNO is a non-invasive biomarker that reflects IL-13-induced nitric oxide production in the airway epithelium [85]. Elevated FeNO (>25 ppb in adults) suggests eosinophilic airway inflammation and predicts responsiveness to inhaled corticosteroids (ICS) and biologics targeting IL-4/IL-13 pathways, such as dupilumab [86]. FeNO also assists in monitoring adherence to anti-inflammatory therapy.

##### Serum Immunoglobulin E (IgE)

Serum IgE is elevated in allergic asthma and indicates sensitization to environmental allergens. While total IgE levels are not always predictive of asthma severity, they are essential for determining eligibility for omalizumab therapy, which is approved for patients with moderate-to-severe allergic asthma and total IgE levels between 30 and 700 IU/mL. Allergen-specific IgE testing further refines diagnosis [87].

##### Periostin

Periostin is a matricellular protein induced by IL-13 in bronchial epithelial cells and serves as a biomarker of chronic T2 inflammation. It has been used in clinical trials to predict responses to anti-IL-13 therapies such as lebrikizumab and tralokinumab. While less commonly used in clinical practice, periostin offers insight into airway remodeling and chronicity of inflammation [88].

#### 2.3.4. Biologics in Asthma Therapeutics

##### Anti-IgE

IgE is 1 of the 5 classes of immunoglobulins (IgM, IgG, IgD, IgA, IgE) primarily involved in T2-high inflammation. It was the last of the immunoglobulin family to be discovered. IgE has a unique chemical structure and is associated with several physiological functions, including hypersensitivity reactions, parasitic infections, and venom protection [89]. Besides, IgE is one of the major players in inflammatory signaling of asthma. As allergens enter the airways, they are presented by antigen-presenting cells to T lymphocytes, which initiate the cell-mediated immune response [8]. Th2 cells and their associated cytokine milieu stimulate B cells to produce IgE antibodies and proallergic cytokines, such as IL-4, IL-5, IL-9, and IL-13. Free IgE released from B cells binds to the high-affinity FCεRI receptor on the surface of mast cells and basophils. The receptor-bound IgE is then cross-linked by an allergen and triggers degranulation and release of prostaglandins, leukotrienes, histamine, proteases, and cytokines, which all lead to the early allergic response [90]. Considering the indispensable role of IgE in instigating inflammatory response, several monoclonal antibodies (mAbs) have been developed to control the inflammatory milieu of asthma via targeting IgE.

##### Omalizumab

Omalizumab was the first mAb approved for use in patients with moderate to severe asthma with evidence of allergic sensitization to perennial aeroallergens. It was co-developed by Genentech and Novartis. Omalizumab is a humanized mAb that binds to the Fc component of free IgE, thereby inhibiting the subsequent binding of IgE to the high-affinity FcεRI receptor on the surfaces of mast cells, basophils, plasmacytoid dendritic cells, and the FcεRII receptor on the surfaces of dendritic cells and eosinophils [91,92]. Several studies have been conducted to evaluate the effectiveness of omalizumab against asthma. Soler M. et al. evaluated omalizumab’s efficacy in moderate-to-severe allergic asthma patients non-responsive to ICS. In this study, during the 7-month trial, patients receiving omalizumab showed 58% fewer exacerbations in the stable-steroid phase and 52% fewer during steroid reduction, despite achieving greater corticosteroid dose reductions than placebo. Besides, omalizumab was well-tolerated with comparable adverse events to placebo. Overall, this study demonstrates that omalizumab safely improves asthma while reducing steroid dependence in the population [93]. Similarly, a pooled analysis of three phase III trials was carried out to evaluate omalizumab’s impact on serious asthma exacerbations with allergic asthma non-responsive to ICS. In this study, omalizumab-treated patients showed significantly lower rates of asthma-related unscheduled outpatient visits, emergency room visits, and hospitalizations compared to placebo. These results demonstrate omalizumab’s efficacy in preventing serious exacerbations and reducing healthcare utilization in moderate-to-severe allergic asthma [94]. Subsequently, Humbert M. et al. showed omalizumab significantly reduced exacerbations and emergency visits in severe persistent asthma patients uncontrolled despite high-dose inhaled corticosteroid (ICS)/long-acting β2-agonist (LABA) therapy. Omalizumab also improved quality of life, lung function, and symptom scores with comparable safety to placebo, demonstrating its efficacy as add-on therapy for this difficult-to-treat population [95]. A close study evaluated omalizumab as add-on therapy in uncontrolled moderate-to-severe allergic asthma patients despite high-dose ICS. Omalizumab significantly reduced annual asthma deterioration incidents by 50% and clinically significant exacerbations by 61%. Omalizumab also improved lung function (forced expiratory volume: FEV1), reduced rescue medication use, and decreased symptom scores, while maintaining a favorable safety profile. These results demonstrate omalizumab’s significant clinical benefits when added to standard therapy in this difficult-to-treat population [96]. Interestingly, a real-world study evaluated omalizumab’s effectiveness in severe allergic asthma patients uncontrolled despite high-dose ICS/LABA therapy. They showed clinically meaningful improvements in asthma control, quality of life, and exacerbation freedom. Significant healthcare utilization reductions were observed versus pretreatment levels, demonstrating omalizumab’s real-world effectiveness exceeding clinical trial results [97]. Parallelly, another real-world study of omalizumab in patients with uncontrolled persistent allergic asthma showed clinically significant exacerbations decreasing from 93.2% pretreatment to 45.9% at 12 months and 32.7% at 24 months. Furthermore, symptoms and rescue medication use halved by 24 months, while maintenance oral corticosteroid (OCS) use dropped from 28.6% (baseline) to 14.2%, demonstrating sustained benefits with a favorable safety profile consistent with clinical trials [98]. A study on mild-to-moderate asthmatics with eosinophilia showed omalizumab significantly reduces serum IgE, airway IgE+ cells, and eosinophils. Whereas, despite these anti-inflammatory effects, airway hyperresponsiveness to methacholine remained unchanged, suggesting IgE/eosinophils may not drive this feature in milder asthma. These findings clarify omalizumab’s mechanism while revealing phenotype-specific treatment effects [99]. An equivalent study investigated omalizumab’s anti-inflammatory mechanisms in allergic asthma patients. They demonstrated significantly increased apoptosis markers (Annexin V+) while reducing GM-CSF+, IL-2+, and IL-13+ lymphocytes without affecting necrosis markers or IL-5/IFN-γ/TNF-α. The results show omalizumab’s dual action in promoting eosinophil apoptosis and suppressing key inflammatory cytokines (IL-2/IL-13). These immunomodulatory effects support omalizumab’s use in allergic asthma control; however, they warrant further mechanistic studies [100]. Another mechanistic study aimed to evaluate omalizumab’s effects on airway remodeling in severe asthma patients. Compared to conventional therapy, omalizumab significantly reduced airway wall thickness and increased luminal area while also decreasing eosinophils and improving FEV1 and asthma quality of life questionnaire (AQLQ) scores. These results propound omalizumab’s potential to reverse airway remodeling while reducing inflammation and improving lung function, though larger long-term studies are needed to confirm these structural benefits [101].

##### Ligelizumab

Ligelizumab is a next-generation high-affinity humanized monoclonal anti-IgE antibody recently developed by Novartis to overcome some of the limitations associated with the clinical use of omalizumab [102]. By binding to the Cε3 domain of IgE, ligelizumab prevents its interaction with the high-affinity FcεRI receptor on mast cells and basophils, leading to reduced degranulation and inflammatory mediator release. This mechanism makes ligelizumab a promising candidate for treating allergic diseases characterized by IgE-mediated inflammation, including chronic spontaneous urticaria (CSU), chronic inducible urticaria (CIndU), food allergies, and asthma [103]. The efficacy of ligelizumab in treating CSU was initially assessed in a phase 2b trial, which aimed to evaluate its potential in patients who were unresponsive to H1-antihistamines. Their findings suggested that ligelizumab could be a more effective alternative to omalizumab in CSU treatment [103]. Following these promising phase 2b results, two phase 3 trials—PEARL 1 and PEARL 2—were conducted to confirm the efficacy and safety of ligelizumab. While both studies met their primary endpoint, demonstrating that ligelizumab was significantly more effective, they failed to show superiority over omalizumab. Due to this lack of differentiation, Novartis decided to halt further development of ligelizumab for CSU [104]. Furthermore, ligelizumab was also investigated in severe asthma. A phase 2b, multicenter, randomized, double-blind, placebo-controlled trial was conducted to assess its efficacy in patients with moderate-to-severe asthma that remained inadequately controlled with standard therapy. In this study, ligelizumab failed to show significant clinical benefits over omalizumab or placebo in improving asthma control, reducing exacerbation rates, or enhancing lung function. These results indicated that ligelizumab did not provide an advantage over existing therapies for moderate-to-severe asthma, leading to a discontinuation of its development in this indication [105]. In addition to CSU and asthma, ligelizumab was evaluated in peanut allergy, a condition where IgE plays a critical role in mediating hypersensitivity reactions. A phase 3, randomized, double-blind study was initiated to assess whether ligelizumab could reduce sensitivity to peanut allergens. However, the study failed to meet its primary endpoints, showing no significant improvement in peanut tolerance among patients receiving ligelizumab. As a result, the trial was terminated, and no further development of ligelizumab for food allergies was pursued [106]. The higher affinity of ligelizumab for IgE and its enhanced IgE suppression suggest that it may still have therapeutic potential in select patient populations. However, its failure to show significant clinical superiority over existing therapies raises questions about its long-term role in allergic disease management. Future research may focus on patient stratification, biomarker identification, or combination approaches to optimize the clinical utility of ligelizumab in IgE-mediated diseases.

##### UB-221

UB-221, developed by United BioPharma, is an anti-IgE mAb of a newer class that is distinct from omalizumab and ligelizumab. In its free form, UB-221 bound abundantly to CD23-occupied IgE, and in oligomeric mAb-IgE complex form, it freely engaged with CD23. On the other hand, ligelizumab reacts limitedly, and omalizumab stays inert toward CD23. These observations are consistent with UB-221 outperforming ligelizumab and omalizumab in CD23-mediated downregulation of IgE production. Besides, UB-221 bound IgE with a strong affinity to prevent FcԑRI-mediated basophil activation and degranulation, exhibiting superior IgE-neutralizing activity to that of omalizumab. In a recent study, UB-221 and ligelizumab bound cellular IgE and effectively neutralized IgE in sera of patients with atopic dermatitis with equal strength, while omalizumab lagged behind [107]. Furthermore, in a phase I, open-label, dose-escalation study, the safety, tolerability, pharmacokinetics, and pharmacodynamics of a single intravenous infusion of UB-221 were evaluated in patients with CSU who were on first-line H1-antihistamine treatment. The study demonstrated that a single UB-221 infusion led to a rapid, dose-dependent reduction in serum free IgE levels, accompanied by significant improvements in disease symptoms, as measured by the Urticaria Activity Score over 7 days (UAS7) [107]. The effects of Ub-221 on asthma and other respiratory diseases are yet to be explored.

##### Anti-IL-4

IL-4 is one of the pivotal cytokines in the development of asthma, particularly in type 2 inflammation. It plays a crucial role in the differentiation of naïve T-helper cells into Th2 cells, which subsequently produce more IL-4, perpetuating the inflammatory response. IL-4 also induces IgE class switching in B cells, leading to IgE production that sensitizes mast cells and basophils, contributing to allergic reactions. Additionally, IL-4 influences eosinophil trafficking to the airways, exacerbating inflammation and airway hyperresponsiveness [65,108]. Given its central role in asthma pathophysiology, IL-4 has been targeted by biologic therapies aiming to mitigate its effects.

##### Dupilumab

Dupilumab is co-developed by Regeneron Pharmaceuticals and Sanofi. It is a fully human mAb targeting the IL-4 receptor alpha (IL-4Rα) and IL-13 and has significantly advanced the treatment landscape for patients with moderate-to-severe asthma, particularly those with type 2 inflammation. By inhibiting IL-4 and IL-13 signaling pathways, dupilumab addresses key mechanisms underlying asthma pathophysiology [109]. Castro M. et al. evaluated the effect of dupilumab in uncontrolled asthma patients. It significantly reduced the annualized rate of severe asthma exacerbations and improved pre-bronchodilator FEV1. Further, this study showed greater benefits of dupilumab in patients with baseline blood eosinophil counts ≥300/mm^3^, with a 65.8% reduction in exacerbations. Although effective, dupilumab instigated adverse events such as blood eosinophilia in 4.1% of dupilumab-treated patients. This transient eosinophilia has been observed in some patients, usually resolving without intervention. Overall, dupilumab demonstrated significant efficacy in reducing exacerbations, improving lung function, and enhancing asthma control, particularly in patients with elevated eosinophils [110]. Rabe K. et al. evaluated the dupilumab effect in patients with glucocorticoid-dependent severe asthma. Interestingly, dupilumab substantially reduced glucocorticoid use by 70.1%. In addition, it also reduced severe exacerbation rates and improved pre-bronchodilator FEV1. Collectively, dupilumab effectively reduced glucocorticoid dependence while improving asthma control and lung function in severe asthma patients [111]. A long-term safety and efficacy of dupilumab study (TRAVERSE) was conducted in moderate-to-severe or oral-corticosteroid-dependent asthma patients who had completed prior dupilumab trials. Over 96 weeks, safety findings were consistent with the known profile, with treatment-emergent adverse events (e.g., nasopharyngitis, injection-site erythema) reported in 76.3–94.7% of patients. Serious adverse events such as asthma exacerbations and pneumonia with four deaths were also reported. Efficacy was sustained, with low annualized exacerbation rates, improved pre-bronchodilator FEV1, and sustained asthma control (ACQ-5) and AQLQ improvements. These results support dupilumab’s long-term safety and efficacy in moderate-to-severe asthma [112]. Furthermore, the TRAVERSE continuation study evaluated the long-term safety of dupilumab in patients with moderate-to-severe asthma who had completed the TRAVERSE study, extending treatment for up to an additional 144 weeks (∼3 years). Dupilumab demonstrated a consistent safety profile, supporting its long-term use in moderate-to-severe asthma [113]. A phase 2b trial evaluated the effect of dupilumab in uncontrolled persistent asthma patients despite being on medium-to-high-dose ICS and LABA. Intriguingly, dupilumab improved lung function and reduced exacerbations, regardless of eosinophil levels, with a favorable safety profile [114]. Similarly, the analysis of the LIBERTY ASTHMA QUEST study evaluated dupilumab’s efficacy in patients with uncontrolled moderate-to-severe asthma, stratified by allergic asthma status. In the allergic asthma subgroup, dupilumab significantly reduced severe exacerbation rates and improved FEV1. Greater efficacy was observed in patients with elevated type 2 biomarkers. Dupilumab also improved asthma control and reduced type 2 inflammatory biomarkers. Similar results were seen in non-allergic asthma patients. These findings underscore dupilumab’s role in addressing IL-4/IL-13-driven inflammation in asthma, regardless of allergic status [115]. The SINUS-24 and SINUS-52 trials evaluated dupilumab’s efficacy in patients with chronic rhinosinusitis with nasal polyps (CRSwNP) and coexisting asthma, stratified by baseline asthma characteristics. Dupilumab significantly improved CRSwNP outcomes and asthma outcomes, regardless of baseline asthma severity or eosinophil levels [116]. Subsequently, the post hoc analysis of the QUEST trial (NCT02414854) evaluated changes in fractional exhaled nitric oxide (FeNO) and blood eosinophil count in dupilumab-treated patients with uncontrolled moderate-to-severe asthma. FeNO levels declined rapidly with dupilumab, while eosinophil levels initially increased but then slightly declined in both groups. However, improvements in pre-bronchodilator FEV1 were inversely associated with FeNO changes and positively associated with eosinophil level changes [117]. These findings suggest biomarker changes may predict lung function benefits but not exacerbation rates.

##### Pascolizumab

Pascolizumab (SB 240683) is a humanized anti-IL-4 mAb developed originally by GlaxoSmithKline and currently in development at Protein Design Laboratories, Inc. Pascolizumab blocks the interaction of IL-4 with its receptor, thereby inhibiting the early events of asthma, including TH2 cell differentiation, eosinophilia, and IgE up-regulation. Preclinical data indicate that blocking these events in vivo may prevent airway inflammatory cell infiltration and remodeling in asthmatic patients [118,119]. In vitro studies demonstrated that a murine mAb 3B9 inhibited IL-4-dependent events, including IL-5 synthesis, TH2 cell activation, and up-regulation of IgE expression. A 3B9 was then humanized (pascolizumab, SB 240683) to reduce immunogenicity in humans. Pascolizumab demonstrated species specificity for both monkey and human IL-4 with no reactivity to mouse, rat, cow, goat, or horse IL-4. In vivo pharmacokinetic and chronic safety testing in cynomolgus monkeys demonstrated that pascolizumab was well tolerated, and no adverse clinical responses occurred after up to 9 months of treatment [118]. A Phase II clinical trial, identified as NCT00024544, was conducted to evaluate the efficacy and safety of pascolizumab in patients with symptomatic, steroid-naive asthma. The study was completed, but development was discontinued due to the observed low efficacy of the treatment in improving asthma symptoms [120].

##### Pitrakinra

Pitrakinra is a recombinant fusion protein developed by Bayer AG as a selective inhibitor of IL-4 and IL-13 signaling. This inhibition disrupts the downstream signaling of these cytokines, effectively dampening the inflammatory processes they trigger. By blocking these cytokine signals, pitrakinra aims to reduce key features of asthma, such as airway inflammation, mucus secretion, and bronchoconstriction [121]. In two phase 2a randomized, double-blind trials, patients with atopic asthma were treated with either pitrakinra or placebo. Study 1 involved subcutaneous injections of pitrakinra, while study 2 used nebulized pitrakinra. In study 1, the maximum decrease in FEV1 was 17.1% in the pitrakinra group versus 23.1% in the placebo group. In study 2, the average FEV1 decrease was 4.4% in the pitrakinra group compared to 15.9% in the placebo group. These results suggest that targeting IL-4 and IL-13 in the lungs could significantly alleviate asthma symptoms, making pitrakinra a promising treatment for allergic asthma [122]. Furthermore, pitrakinra was investigated in an ICS withdrawal in uncontrolled, moderate to severe asthma patients. Efficacy was not demonstrated in the overall study population, suggesting additional mechanisms beyond IL/IL-13 pathways might predominate in some of the patients in this heterogeneous population of uncontrolled asthma. However, significant efficacy was observed in certain pre-specified subpopulations, such as (1) eosinophilic asthma, (2) upper tertile FE NO, and (3) patients with the GG homogenous allele of the rs8832 SNP of the IL4. Additionally, significant improvements in symptom scores and/or spirometry were observed in some of the responder subgroups. Overall, they concluded that Pitrakinra, by blocking IL-4R, demonstrated clinical efficacy within defined subpopulations of uncontrolled asthma [123].

##### Anti-IL-5

IL-5 is a key cytokine involved in the pathophysiology of asthma, particularly in the context of eosinophilic inflammation. Eosinophils play a central role in the chronic inflammation observed in many asthma patients, contributing to airway hyperresponsiveness, mucus production, and tissue damage [62]. Targeting IL-5 has become an important therapeutic strategy in asthma, particularly for patients with severe eosinophilic asthma who are not adequately controlled by conventional therapies like ICS or bronchodilators [124].

##### Mepolizumab

Mepolizumab, developed by GlaxoSmithKline, is an mAb that targets and inhibits IL-5. By blocking IL-5, mepolizumab reduces the recruitment and activation of eosinophils in the airway, which can alleviate inflammation and reduce asthma exacerbations [125]. The efficacy of mepolizumab has been evaluated in various clinical trials, demonstrating significant improvements in asthma control, particularly in patients with severe, eosinophilic asthma. Haldar P. et al. evaluated the efficacy of mepolizumab in reducing exacerbations in refractory eosinophilic asthma patients. Mepolizumab significantly reduced the number of severe exacerbations, improved AQLQ scores, and significantly decreased eosinophil counts in both blood and sputum. However, there were no significant differences between groups in asthma symptoms, FEV1 after bronchodilator use, or airway hyperresponsiveness. These findings suggest that mepolizumab effectively reduces exacerbations and improves quality of life in patients with refractory eosinophilic asthma, highlighting the role of eosinophils as key effector cells in severe asthma exacerbations [126]. The dose-ranging efficacy and safety of mepolizumab in patients with severe asthma (DREAM) was a pivotal Phase 2b trial that assessed the efficacy of mepolizumab in patients with severe asthma and elevated eosinophil levels. The study found that mepolizumab significantly reduced the annual rate of asthma exacerbations. Additionally, patients receiving mepolizumab demonstrated improved asthma control and quality of life. The safety profile of mepolizumab was favorable, with no unexpected adverse events [127]. Subsequently, the MENSA study investigated mepolizumab in patients with severe eosinophilic asthma who continued to experience exacerbations despite high-dose inhaled glucocorticoid therapy. The results showed a significant reduction in exacerbations with mepolizumab compared to placebo. Lung function improved, with a greater increase in FEV1 in the treatment groups. AQLC and asthma control measures also showed meaningful improvements. Besides, the safety profile of mepolizumab was comparable to placebo. These findings confirm that mepolizumab is an effective treatment option for severe eosinophilic asthma, reducing exacerbations and improving disease control [125]. The post-hoc analysis examined the relationship between baseline blood eosinophil counts and the efficacy of mepolizumab in severe eosinophilic asthma patients. Data from two randomized, double-blind, placebo-controlled trials (DREAM and MENSA) were analyzed. Mepolizumab significantly reduced the annual exacerbation rate (AER). Further analysis showed that the efficacy of mepolizumab increased with higher baseline eosinophil counts. These findings highlight the importance of baseline eosinophil levels as a biomarker to identify patients who are most likely to benefit from mepolizumab therapy [128]. Bel E.H. et al. assessed the glucocorticoid-sparing effect of mepolizumab in severe eosinophilic asthma patients who required daily oral glucocorticoids for asthma control. Mepolizumab significantly increased the likelihood of glucocorticoid dose reduction, with a 2.39 times greater probability compared to placebo. The median reduction in glucocorticoid dose was 50% in the mepolizumab group. Despite reduced glucocorticoid use, the mepolizumab group had a 32% lower AER and improved asthma control, with a 0.52-point reduction on the AQLC. The safety profile of mepolizumab was similar to placebo. These findings demonstrate that mepolizumab enables patients with severe eosinophilic asthma to reduce their dependence on systemic glucocorticoids while maintaining better asthma control and reducing exacerbations [129].

##### Reslizumab

Reslizumab is a humanized monoclonal IL-5 antibody developed by Teva Pharmaceuticals that has been approved in the USA for patients aged ≥18 years as add-on maintenance treatment for severe asthma with an eosinophilic phenotype [130]. Castro M. et al. conducted a phase 3 trial in uncontrolled asthma patients on ICS and showed reslizumab significantly reduces exacerbations. Besides, adverse events were comparable, with asthma worsening, upper respiratory infections, and nasopharyngitis being most common [131]. Subsequently, another study evaluated the efficacy and safety of reslizumab in poorly controlled asthma patients. While no significant difference in FEV1 was observed in the overall population or in those with eosinophils < 400 cells/μL, reslizumab significantly improved lung function and symptom control in patients with eosinophils ≥ 400 cells/μL. Reslizumab was well tolerated, with fewer adverse events compared to placebo [132]. Similarly, Ibrahim H. et al. assessed the safety and clinical efficacy of reslizumab in severe eosinophilic asthma patients. Reslizumab, after 1 year of treatment, significantly improved asthma control, with 35.7% of steroid-dependent patients discontinuing steroids and a 79% reduction in exacerbations. By 2 years, the exacerbation reduction was 88%. Reslizumab was well tolerated, with only one patient discontinuing due to side effects. These findings confirm the efficacy of anti-IL5 therapy in severe asthma with eosinophilic inflammation [133]. Furthermore, a real-world observational study evaluated the effectiveness of reslizumab against severe eosinophilic asthma. The study found that reslizumab significantly reduced asthma exacerbations, maintenance OCS use, and maintenance dose, with similar outcomes in both biologic-naive patients and those switching from another type 2 biologic. The overall treatment response was rated as good or excellent in 59.2% of patients. Physicians also reported the added value of reslizumab after switching biologics. These findings confirm reslizumab’s effectiveness in reducing exacerbations and corticosteroid use in severe eosinophilic asthma [134].

##### Benralizumab

Benralizumab, developed by MedImmune and AstraZeneca, is a humanized, afucosylated (engineered to eliminate fucose sugars from the oligosaccharides in the Fc region) monoclonal antibody targeted against the alpha subunit of the IL-5 receptor that induces direct, rapid, and nearly complete depletion of eosinophils by means of natural killer cell-mediated antibody-dependent cellular cytotoxic effects [135]. Bleecker E.R. et al., in a phase 3 study, evaluated the safety and efficacy of benralizumab in patients with severe, uncontrolled asthma with high-dose ICS/LABA and eosinophilic asthma. Benralizumab significantly reduced AERs and improved prebronchodilator FEV1. Common adverse events included worsening asthma and nasopharyngitis [136]. A similar study showed that benralizumab significantly reduces AER and improves FEV1 and asthma symptom scores. The treatment was generally well tolerated, with nasopharyngitis and worsening asthma being the most common adverse events. Overall, these results support benralizumab as an effective therapy for severe eosinophilic asthma patients [137]. Furthermore, a phase 3 extension study assessed benralizumab’s long-term safety and efficacy. In this study, common adverse events included viral upper respiratory tract infection and worsening asthma, while serious adverse events such as pneumonia were rare. Besides, the adverse event rates were consistent between benralizumab and placebo groups and aligned with previous trials, with no new safety concerns emerging from long-term eosinophil depletion. These findings confirm benralizumab’s sustained safety and efficacy over two years, supporting its use for long-term management of severe eosinophilic asthma [138]. Subsequently, FitzGerald J.M., et al. conducted a pooled analysis of the SIROCCO and CALIMA phase 3 studies to evaluate the benralizumab efficacy across different baseline blood eosinophil thresholds and exacerbation histories. Benralizumab reduced the AER by 36% in patients with ≥0 eosinophils/μL. Greater reductions in AER were observed with higher eosinophil thresholds and more frequent exacerbation histories. These findings highlight benralizumab’s efficacy, particularly in patients with elevated eosinophils and frequent exacerbations [139]. Nair P. et al. evaluated Benralizumab for its oral glucocorticoid-sparing effects in severe eosinophilic asthma patients reliant on oral glucocorticoids. Benralizumab significantly reduced median oral glucocorticoid doses by 75% compared to 25% with placebo, with patients 4 times more likely to achieve dose reductions. Exacerbation rates were 55% to 70% lower compared to placebo. However, no significant improvement in FEV1 was observed, and effects on asthma symptoms were mixed. Adverse event rates were similar across groups [140]. A recent study by Ramakrishnan S. et al. demonstrated that subcutaneous injection of Benralizumab can be used for acute eosinophilic exacerbations with better outcomes than the current standard therapy of prednisolone alone. Their results offer a novel treatment regimen for eosinophilic asthma and COPD [141]. Overall, benralizumab demonstrated clinically relevant benefits in reducing glucocorticoid use and exacerbations, supporting its use in severe eosinophilic asthma.

##### Depemokimab

Depemokimab, a next-generation biologic with high binding affinity for IL-5, is being developed by GSK as an ultra-long-acting therapy that may allow dosing just twice a year. In the phase 3 SWIFT-1 and SWIFT-2 trials, patients with severe eosinophilic asthma who received depemokimab showed a significant reduction in asthma exacerbations over 52 weeks compared to placebo. The annualized exacerbation rate dropped by over 50% in both trials. While no major improvements were seen in quality-of-life scores (SGRQ), the treatment was generally well tolerated, with similar rates of adverse events in both groups. These findings support depemokimab as a promising option for long-term control in eosinophilic asthma [142].

##### Anti-IL-13

IL-13 is an immunoregulatory cytokine secreted predominantly by activated Th-2 cells and is involved in Type 2 inflammation. Over the past several years, it has become evident that IL-13 is a key mediator in the pathogenesis of allergic airway inflammation, and thereby it has been identified as a possible therapeutic target in the treatment of asthma [4,143,144]. Several biologics targeting IL-13 have been developed for the treatment of asthma, particularly for patients with type 2 inflammation.

##### Lebrikizumab

Lebrikizumab is an IgG4 humanized mAb that binds to IL-13. The molecule was developed by Genentech. The stability of lebrikizumab has been increased by a single joint mutation on the hinge portion of the molecule [145]. Corren J. et al. evaluated lebrikizumab in uncontrolled asthma patients despite being on ICS. The lebrikizumab treatment showed a greater improvement in FEV1 compared to placebo, with even greater benefits in the high-periostin subgroup versus the low-periostin subgroup. However, lebrikizumab was associated with increased musculoskeletal side effects [146]. At the same time, a phase II study evaluated the efficacy and safety of lebrikizumab in asthmatic patients not receiving ICS. While all lebrikizumab dose groups showed higher FEV1 improvements compared to placebo, the differences were neither statistically nor clinically significant. No meaningful FEV1 changes were observed between periostin subgroups. However, lebrikizumab significantly reduced the risk of treatment failure across all doses versus placebo, with consistent results across periostin subgroups and no dose-dependent differences. In addition, lebrikizumab was well tolerated. This study suggested that while IL-13 blockade alone may not improve lung function in this population, it may enhance disease control by preventing treatment failure [147]. Furthermore, the LUTE and VERSE studies evaluated lebrikizumab in uncontrolled asthma patients despite being on medium-to-high-dose ICS. Pooled data showed lebrikizumab reduced exacerbation rates, particularly in periostin-high patients (60% reduction) compared to periostin-low patients (5% reduction), with no dose-response relationship. Lung function improvements were also greater in periostin-high patients versus periostin-low patients. Besides, brikizumab was well tolerated, with no significant safety concerns. These findings support lebrikizumab’s efficacy in reducing exacerbations and improving lung function in uncontrolled asthma patients, particularly those with high periostin levels, extending previous evidence of its therapeutic benefits [148]. Scheerens H. et al. evaluated the effect of lebrikizumab in subjects with mild asthma undergoing bronchial allergen challenge. Lebrikizumab reduced late asthmatic response (LAR) by 48% compared to placebo, though this was not statistically significant. Exploratory analyses suggested greater LAR reduction in subjects with elevated baseline eosinophils, IgE, or periostin levels. Lebrikizumab also reduced systemic Th2 inflammation markers, including IgE, CCL13, and CCL17, by approximately 25%. Additionally, the treatment was well tolerated. These findings indicate lebrikizumab’s potential to attenuate allergen-induced airway responses in mild asthma, particularly in patients with elevated Th2 biomarkers [149]. Piper E. et al. evaluated tralokinumab in moderate-to-severe uncontrolled asthma. The ACQ-6 score showed no significant improvement for tralokinumab versus placebo. However, tralokinumab improved pre-bronchodilator FEV1 with a dose-response trend and reduced rescue β2-agonist use. Besides, tralokinumab was well tolerated, with no serious adverse events [150]. Furthermore, a study evaluated lebrikizumab in two replicate phase 3 trials (LAVOLTA I and II) involving uncontrolled asthma despite being on ICS. In LAVOLTA I, lebrikizumab significantly reduced exacerbation rates in biomarker-high patients. In LAVOLTA II, reductions were observed but did not reach statistical significance. Additionally, pooled data showed similar rates of treatment-emergent adverse events, serious adverse events, and discontinuations between lebrikizumab and placebo. While lebrikizumab did not consistently reduce exacerbations, it demonstrated IL-13 blockade through pharmacodynamic biomarkers, suggesting potential clinical relevance [151]. Subsequently, the post hoc analysis of the LAVOLTA I, II, and ACOUSTICS trials evaluated lebrikizumab in a subpopulation of patients with uncontrolled asthma, elevated blood eosinophils (≥300 cells/μL), and a history of exacerbations. In adults, lebrikizumab significantly reduced AER by 38% to 41% compared to placebo. In adolescents, reductions were 59% to 64%. Similar benefits were observed in patients with elevated FeNO and prior exacerbations. Adverse events were mostly mild to moderate, with few leading to discontinuation. Overall, these findings suggest lebrikizumab effectively reduces exacerbations in patients with elevated eosinophils, FeNO, and a history of exacerbations, highlighting its potential in this well-defined subpopulation [152].

##### Tralokinumab

Tralokinumab is a fully human IgG4 monoclonal antibody that specifically binds to IL-13 with high affinity. Blocking interaction with the IL-13 receptor inhibits downstream IL-13 signaling [153]. This was developed by AstraZeneca. Brightling C.E. et al. evaluated tralokinumab in patients with severe uncontrolled asthma. The AER showed no significant difference between tralokinumab and placebo. However, tralokinumab significantly improved FEV1. Besides, post-hoc analyses suggested potential benefits in subgroups, such as patients with FEV1 reversibility ≥12% and elevated biomarkers (DPP-4, periostin), showing improvements in exacerbation rates, FEV1, and asthma control. While tralokinumab did not reduce exacerbation rates overall, subgroup findings indicate potential efficacy in specific populations, warranting further investigation in phase 3 trials [154]. The STRATOS 1 and STRATOS 2 phase 3 trials evaluated the effect of tralokinumab in severe uncontrolled asthma patients. In STRATOS 1, tralokinumab did not significantly reduce AER in the overall population but showed a 44% reduction in patients with baseline FENO ≥37 ppb. However, in STRATOS 2, tralokinumab did not significantly reduce AER in the FENO-high population. These inconsistent results suggest IL-13 may not play a central role in severe asthma exacerbations, limiting tralokinumab’s efficacy in this population [155]. Furthermore, Busse W.W. et al. assessed tralokinumab for its OCS-sparing potential in severe, uncontrolled asthma patients requiring maintenance OCS and ICSs/LABAs. The percentage reduction in OCS dose showed no significant difference between tralokinumab and placebo. Secondary endpoints, including the proportion of patients achieving OCS doses ≤5 mg or ≥50% reduction and asthma exacerbation rates, also showed no significant differences. In addition, adverse events were similar between groups, though upper respiratory tract infections were more common with tralokinumab. Overall, tralokinumab did not demonstrate significant OCS-sparing effects in severe asthma patients [156].

##### Tezepelumab

Tezepelumab is a human monoclonal antibody that blocks thymic stromal lymphopoietin (TSLP), an upstream epithelial cytokine involved in asthma pathogenesis. It is developed by AstraZeneca. In the phase 2 PATHWAY trial, tezepelumab significantly reduced asthma exacerbations by 62–71% across all dose groups compared to placebo over 52 weeks, with benefits observed regardless of baseline eosinophil counts. Improvements in pre-bronchodilator FEV₁ were also noted, and the treatment was well tolerated [157]. Building on these findings, the phase 3 NAVIGATOR trial demonstrated that tezepelumab reduced exacerbations by over 55% and improved lung function, asthma control, and quality of life in patients with severe, uncontrolled asthma. Importantly, efficacy was maintained even in patients with low eosinophil counts (<300 cells/μL) [158]. A pooled analysis of PATHWAY and NAVIGATOR further confirmed that tezepelumab led to a 60% reduction in annualized asthma exacerbations and improved secondary outcomes across both type 2–high and type 2–low subgroups. The safety profile was comparable to placebo in all studies, highlighting tezepelumab’s broad potential across diverse asthma phenotypes [159].

#### 2.3.5. Emerging Biologics for Type 1 Inflammation

While most of the biologics (mAb) for asthma target type 2 inflammation, recent studies have explored the type 1 inflammation (Th1 high asthma). TNF-α and IFN-γ are two major pro-inflammatory cytokines associated with type 1 inflammation [4]. Among the two, TNF-α is a potential target for asthma treatment, especially severe asthma. However, controlled studies have shown controversial results, and the risk-benefit profile of TNF-blocking agents is still debated [160]. Besides, currently no biologics targeting IFNγ for asthma have been developed, which leaves this door completely open for research.

Infliximab is a purified, recombinant DNA-derived chimeric IgG mAb that contains both murine and human components designed to inhibit TNF-α [161]. It was developed by Janssen Biotech. A case series by Taillé C. et al. evaluated infliximab efficacy in severe, steroid-dependent, refractory asthma patients. All patients had frequent exacerbations and hospitalizations despite maximal inhaled therapy, oral steroids, and omalizumab. Six patients treated with infliximab for ≥3 months showed improved asthma control, with four discontinuing OCS and reduced exacerbations/hospitalizations, particularly in brittle asthma cases. However, two patients experienced severe adverse events (bacterial pneumonia, melanoma progression). Besides, three patients continued infliximab for >2 years with good tolerance. The findings suggest infliximab may benefit a subset of severe refractory asthma patients, with a favorable risk-benefit profile, though controlled trials are needed [162].

Golimumab is another monoclonal antibody developed by Janssen Biotech that binds to both membrane-bound and soluble TNF-α [163]. Wenzel S.E. et al. evaluated the golimumab effect in severe, uncontrolled asthma patients despite high-dose ICSs and LABAs. No significant improvements were observed in prebronchodilator FEV1 or severe exacerbation rates through Week 24. Discontinuation rates were higher with golimumab (19.5%) than with placebo (2.6%), and serious adverse events, including infections and malignancies, were more frequent in golimumab-treated patients. One death occurred in the active treatment group. Overall, the study concluded that golimumab did not demonstrate a favorable risk-benefit profile in severe persistent asthma, leading to early discontinuation of treatment [164].

Adalimumab is an mAb that inhibits TNF-α [165]. It was developed by AbbVie. Catal F. et al. evaluated the anti-TNF-α effect of adalimumab in a murine asthma model. It significantly reduced lung damage, inflammatory cell infiltration around bronchi, alveolar wall inflammation, and alveolar wall thickness compared to the untreated group. In addition, adalimumab also decreased peribronchial smooth muscle hypertrophy and edema. The findings suggest adalimumab improves lung histology and reduces inflammation in acute asthma, highlighting its potential therapeutic role [166]. Further study on humans is warranted.

Etanercept (ENT) is a soluble receptor that binds both TNF-α and TNF-β to inhibit the inflammatory response. It was developed by Immunex Corporation. ENT is commonly used to control ankylosing spondylitis, juvenile idiopathic arthritis, plaque psoriasis, psoriatic arthritis, and rheumatoid arthritis [167]. A phase 2 trial evaluated the effect of etanercept in moderate-to-severe persistent asthma patients. The primary endpoint FEV1 showed no significant difference between ETN and placebo. Secondary endpoints, including peak expiratory flow, asthma control, exacerbations, and quality of life, also showed no improvement with ETN. However, ETN was well-tolerated with no unexpected safety issues. The study concluded that ETN did not demonstrate clinical efficacy in this population over 12 weeks, but longer-term studies in specific asthma subsets may be warranted [168].

#### 2.3.6. Emerging Biologics for Type 17 Inflammation

Type 17 inflammation, or inflammation mediated by Th17 cells and their cytokine IL-17, is a key driver of chronic inflammation, asthma, and autoimmune diseases, including psoriasis, rheumatoid arthritis, and inflammatory bowel disease [169]. mAbs have been developed against IL-17 to curb asthma and other diseases.

Secukinumab, developed by Novartis, is a recombinant high-affinity fully human monoclonal IL-17A antibody of the IgG1κ isotype, which binds to human IL-17A and neutralizes its bioactivity [170]. A recent study evaluated the effects of secukinumab in a murine model combining ovalbumin-induced asthma with LPS-triggered acute lung injury, a scenario mimicking severe neutrophilic inflammation. Secukinumab effectively modulated immune responses by significantly reducing IL-17 levels, similar to standard therapy dexamethasone (DEXA). While DEXA broadly increased Th2 cytokines (IL-4, IL-5, and IL-13) and TNF-α (Th1), secukinumab selectively suppressed IL-5 without altering TNF-α, suggesting a more targeted anti-inflammatory profile. Both treatments decreased IFN-γ and IL-6. The findings highlight secukinumab’s potential to attenuate Th17-mediated neutrophilic inflammation in asthma-ALI overlap, offering a complementary mechanism to steroids [171]. Similarly, a phase II study evaluated whether secukinumab could reduce ozone-induced airway neutrophilia. Results showed no significant reduction in sputum neutrophils with secukinumab compared to placebo. The negative findings suggest the ozone challenge model may not fully replicate IL-17-driven inflammation or that higher secukinumab doses are needed. These results indicate IL-17A inhibition alone may be insufficient for acute neutrophilic inflammation in this model, warranting further investigation with alternative challenges or modified dosing regimens. In addition, these studies provide important insights into the limitations of current models for testing IL-17-targeted therapies in neutrophilic airway diseases [172].

Brodalumab is a human IgG2 monoclonal antibody targeting IL-17RA, which is currently registered for the treatment of psoriasis vulgaris, psoriatic arthritis, pustular psoriasis, and psoriatic erythroderma [173]. It was developed by Amgen. A phase II study evaluated the efficacy and safety of brodalumab in patients with moderate-to-severe asthma inadequately controlled by ICSs. The results showed no significant treatment benefit of brodalumab over placebo in the overall study population. However, a prespecified subgroup analysis of patients with high bronchodilator reversibility (post-bronchodilator FEV1 improvement ≥20%) demonstrated a nominally significant improvement in ACQ score. Safety profiles were comparable across treatment groups, with the most common adverse events being asthma exacerbations, upper respiratory infections, and injection site reactions. These findings suggest that while IL-17 receptor blockade does not provide broad clinical benefit in unselected asthma populations, there may be a potential role for brodalumab in specific subgroups characterized by high bronchodilator reversibility, needing further investigation in biomarker-defined patient populations [174].

Ixekizumab is an IgG4 mAb that selectively targets IL-17A with high affinity [175]. It was developed by Eli Lilly and Company. Studies have shown the safety of ixekizumab in adult patients with moderate-to-severe psoriasis [176,177]. However, studies on asthma are obscure. One case study reported concomitant use of mepolizumab and ixekizumab in a patient with severe eosinophilic asthma [178]; however, it needs further and in-depth elaboration.

Emerging strategies such as stem cell-based therapies, CRISPR-mediated gene editing, and regenerative medicine hold promise for precision treatment [179,180,181]. Additionally, novel delivery systems like nanoparticles and hydrogels are being explored to enhance drug targeting [182,183]. Long non-coding RNAs (lncRNAs) are also being investigated for their role in asthma pathogenesis and potential as therapeutic targets [184].

## 3. Conclusions

**Figure 1:**
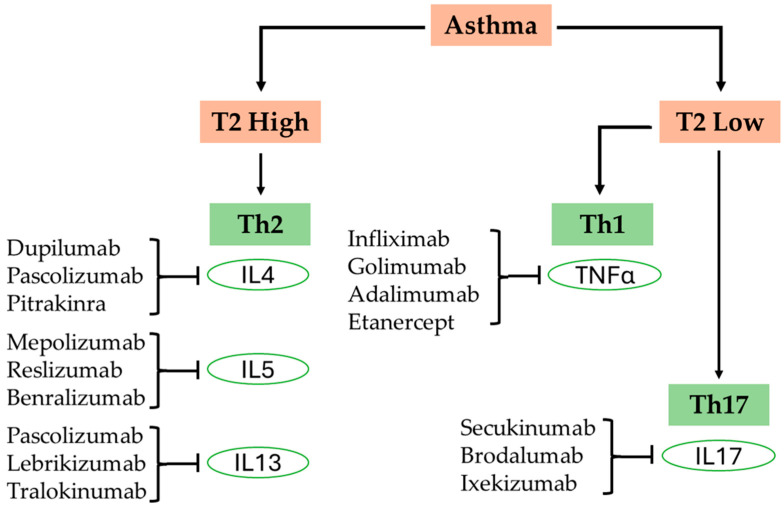
A schematic of asthma endotypes, associated inflammation, and their treatments.

Asthma is a heterogenic and complex immune-mediated disease with inflammation at its core. Advancement in understanding about immunologic mechanisms involving contributions of Th2, Th1, and Th17 cells has substantially changed our treatment strategies. Targeted therapies against Type 2 inflammation (Figure 1 and Table 2), such as anti-IgE, anti-IL-5, and anti-IL-4/IL-13 therapies, have greatly improved outcomes of treatment in moderate-to-severe uncontrolled asthmatic patients despite conventional therapy. These treatments not only lowered rates of exacerbation and improved lung function but also improved quality of life in many patients. Despite this, there remains a significant unmet need in patients with T2-low, neutrophilic, or steroid-resistant asthma, for whom currently available biologics are mainly ineffective. New biologics now attempt to bridge the gap. New drugs such as TNF-α inhibitors (e.g., infliximab, golimumab) and IL-17 inhibitors (e.g., secukinumab) are promising in their capacity to address T1 and T17 inflammation (Figure 1 and Table 3), although concerns such as variable clinical response and safety remain. Furthermore, newer-generation drugs such as UB-221 and combination biologics are under investigation to provide broader and more sustained control of inflammation. Personalized medicine, based on biomarkers like eosinophil count, FeNO, and periostin, will be critical in optimizing biologic use maximally and getting patients on the best therapy for their specific endotype. Despite their benefits, biologics face challenges including high cost, need for parenteral administration, limited accessibility, long-term safety concerns, and heterogeneity in patient response. As research advances, the future of asthma treatment lies in precision immunotherapy, tailoring biologics to address the unique immune profile of each patient. Additional research on novel targets, strategies of enhanced stratification tools, and safety assessments over the long term will enable the extension of the use of biologics to the wider asthma population, especially those with non-T2 asthma.

## Figures and Tables

**Table 2:** Summary of biologics used in T2-high asthma.

Biologics	Target	Indication	Approval	Developer	References
Omalizumab	IgE	Moderate-to-severe allergic asthma	Approved	Genentech and Novartis	[93,94,95,96,97,98,99,100,101]
Ligelizumab	IgE	Investigational	Discontinued	Novartis	[102,103,104]
UB-221	IgE	Investigational	Unknown	United BioPharma	[107]
Dupilumab	IL-4	Moderate-to-severe eosinophilic or OCS-dependent asthma	Approved	Regeneron pharmaceuticals and Sanofi	[110,111,112,113,114,115,116,117]
Pascolizumab	IL-4 and IL-13	Investigational	Unknown	GlaxoSmithKline and Protein Design Laboratories	[118,119,120]
Pitrakinra	IL-4	Atopic and uncontrolled asthma	Discontinued	Bayer AG	[121,122,123]
Mepolizumab	IL-5	Severe eosinophilic asthma	Approved	GlaxoSmithKline	[125,126,127,128,129]
Reslizumab	IL-5	Severe eosinophilic asthma	Approved	Teva Pharmaceuticals	[130,131,132,133,134]
Benralizumab	IL-5	Severe eosinophilic asthma	Approved	MedImmune, AstraZeneca	[135,136,137,138,139,140]
Lebrikizumab	IL-13	Severe eosinophilic asthma	Discontinued	Genentech	[145,146,147,148,149,150,151,152]
Tralokinumab	IL-13	Severe eosinophilic asthma	Discontinued	AstraZeneca	[153,154,155,156]

**Table 3:** Emerging biologics for asthma.

Biologics	Target	Indication	Approval	Developer	References
Infliximab	TNF-α	Severe, steroid-dependent, refractory asthma	Case series	Janssen Biotech	[161,162]
Golimumab	TNF-α	Severe, uncontrolled asthma	Discontinued	Janssen Biotech	[163,164]
Adalimumab	TNF-α	Investigational	Preclinical (murine)	AbbVie	[165,166]
Etanercept	TNF-α	Moderate-to-severe persistent asthma	Phase II	Immunex Corporation	[167,168].
Secukinumab	IL-17	Airway neutrophilia	Phase II	Novartis	[170,171,172]
Brodalumab	IL-17	Moderate-to-severe asthma	Phase II	Amgen	[173,174]
Ixekizumab	IL-17	Severe Eosinophilic Asthma	Case report	Eli Lilly and Company	[175,178]

## References

[1] Prakash Y.S. (2020). Asthma without Borders. Am. J. Physiol.-Lung Cell. Mol. Physiol..

[2] Ambhore N.S., Balraj P., Kumar A., Reza M.I., Ramakrishnan Y.S., Tesch J., Lohana S., Sathish V. (2024). Kiss1 Receptor Knockout Exacerbates Airway Hyperresponsiveness and Remodeling in a Mouse Model of Allergic Asthma. Respir. Res..

[3] Balraj P., Ambhore N.S., Ramakrishnan Y.S., Borkar N.A., Banerjee P., Reza M.I., Varadharajan S., Kumar A., Pabelick C.M., Prakash Y.S. (2024). Kisspeptin/KISS1R Signaling Modulates Human Airway Smooth Muscle Cell Migration. Am. J. Respir. Cell Mol. Biol..

[4] Ford M.L., Reza M.I., Ruwanpathirana A., Sathish V., Britt R.D. (2025). Integrative Roles of Pro-Inflammatory Cytokines on Airway Smooth Muscle Structure and Function in Asthma. Immunol. Rev..

[5] Murdoch J.R., Lloyd C.M. (2010). Chronic Inflammation and Asthma. Mutat. Res..

[6] Kuruvilla M.E., Lee F.E.-H., Lee G.B. (2019). Understanding Asthma Phenotypes, Endotypes, and Mechanisms of Disease. Clin. Rev. Allergy Immunol..

[7] Holgate S.T. (2012). Innate and Adaptive Immune Responses in Asthma. Nat. Med..

[8] Wenzel S.E. (2012). Asthma Phenotypes: The Evolution from Clinical to Molecular Approaches. Nat. Med..

[9] Xie C., Yang J., Gul A., Li Y., Zhang R., Yalikun M., Lv X., Lin Y., Luo Q., Gao H. (2024). Immunologic Aspects of Asthma: From Molecular Mechanisms to Disease Pathophysiology and Clinical Translation. Front. Immunol..

[10] Desai M., Oppenheimer J. (2016). Elucidating Asthma Phenotypes and Endotypes: Progress towards Personalized Medicine. Ann. Allergy Asthma Immunol..

[11] Akar-Ghibril N., Casale T., Custovic A., Phipatanakul W. (2020). Allergic Endotypes and Phenotypes of Asthma. J. Allergy Clin. Immunol. Pract..

[12] Reza M.I., Balraj P., Ramakrishnan Y., Pabelick C.M., Prakash Y.S., Britt R.D., Sathish V. (2023). Activation of Aryl Hydrocarbon Receptor Inhibits Airway Smooth Muscle Proliferation. A70. Relax Already! Mechanistic Insights into Airway Smooth Muscle Biology.

[13] Reza M.I., Kumar A., Balraj P., Pabelick C.M., Prakash Y.S., Britt R.D., Sathish V. (2024). Activation of Aryl Hydrocarbon Receptor Inhibits Extracellular Matrix in Airway Smooth Muscle Cells. B79. Airway Smooth Muscle in Asthma.

[14] Ambhore N.S., Balraj P., Pabelick C.M., Prakash Y.S., Sathish V. (2024). Estrogen Receptors Differentially Modifies Lamellipodial and Focal Adhesion Dynamics in Airway Smooth Muscle Cell Migration. Mol. Cell. Endocrinol..

[15] Ambhore N.S., Kalidhindi R.S.R., Pabelick C.M., Hawse J.R., Prakash Y.S., Sathish V. (2019). Differential Estrogen-Receptor Activation Regulates Extracellular Matrix Deposition in Human Airway Smooth Muscle Remodeling via NF-κB Pathway. FASEB J..

[16] Borkar N.A., Ambhore N.S., Kalidhindi R.S.R., Pabelick C.M., Prakash Y.S., Sathish V. (2022). Kisspeptins Inhibit Human Airway Smooth Muscle Proliferation. JCI Insight.

[17] Louis R., Lau L.C., Bron A.O., Roldaan A.C., Radermecker M., Djukanović R. (2000). The Relationship between Airways Inflammation and Asthma Severity. Am. J. Respir. Crit. Care Med..

[18] Gillissen A., Paparoupa M. (2015). Inflammation and Infections in Asthma. Clin. Respir. J..

[19] Visca D., Ardesi F., Zappa M., Pignatti P., Grossi S., Vanetti M., Migliori G.B., Centis R., Angeli F., Spanevello A. (2024). Asthma and Hypertension: The Role of Airway Inflammation. Front. Med..

[20] Chandrasekara S., Wark P. (2024). Biologic Therapies for Severe Asthma with Persistent Type 2 Inflammation. Aust. Prescr..

[21] McGregor M.C., Krings J.G., Nair P., Castro M. (2019). Role of Biologics in Asthma. Am. J. Respir. Crit. Care Med..

[22] Kaur R., Chupp G. (2019). Phenotypes and Endotypes of Adult Asthma: Moving toward Precision Medicine. J. Allergy Clin. Immunol..

[23] Gauthier M., Kale S.L., Ray A. (2025). T1-T2 Interplay in the Complex Immune Landscape of Severe Asthma. Immunol. Rev..

[24] Ray A., Kolls J.K. (2017). Neutrophilic Inflammation in Asthma and Association with Disease Severity. Trends Immunol..

[25] Spellberg B., Edwards J.E. (2001). Type 1/Type 2 Immunity in Infectious Diseases. Clin. Infect. Dis..

[26] Hammad H., Lambrecht B.N. (2021). The Basic Immunology of Asthma. Cell.

[27] Fahy J.V., Jackson N.D., Sajuthi S.P., Pruesse E., Moore C.M., Everman J.L., Rios C., Tang M., Gauthier M., Wenzel S.E. (2023). Asthma Severity and Corticosteroid Response Depend on Variable Type 1 and Type 2 Inflammation in the Airway. medRxiv.

[28] Luo W., Hu J., Xu W., Dong J. (2022). Distinct Spatial and Temporal Roles for Th1, Th2, and Th17 Cells in Asthma. Front. Immunol..

[29] Kardas G., Kuna P., Panek M. (2020). Biological Therapies of Severe Asthma and Their Possible Effects on Airway Remodeling. Front. Immunol..

[30] Olsthoorn S.E.M., van Krimpen A., Hendriks R.W., Stadhouders R. (2025). Chronic Inflammation in Asthma: Looking Beyond the Th2 Cell. Immunol. Rev..

[31] Wang P., Wang Y., Xie L., Xiao M., Wu J., Xu L., Bai Q., Hao Y., Huang Q., Chen X. (2019). The Transcription Factor T-Bet Is Required for Optimal Type I Follicular Helper T Cell Maintenance During Acute Viral Infection. Front. Immunol..

[32] Szabo S.J., Kim S.T., Costa G.L., Zhang X., Fathman C.G., Glimcher L.H. (2000). A Novel Transcription Factor, T-Bet, Directs Th1 Lineage Commitment. Cell.

[33] Annunziato F., Cosmi L., Liotta F., Maggi E., Romagnani S. (2014). Human Th1 Dichotomy: Origin, Phenotype and Biologic Activities. Immunology.

[34] Brightling C., Berry M., Amrani Y. (2008). Targeting TNF-α: A Novel Therapeutic Approach for Asthma. J. Allergy Clin. Immunol..

[35] Nam H.-S., Lee S.Y., Kim S.J., Kim J.S., Kwon S.S., Kim Y.K., Kim K.H., Moon H.S., Song J.S., Park S.H. (2009). The Soluble Tumor Necrosis Factor-Alpha Receptor Suppresses Airway Inflammation in a Murine Model of Acute Asthma. Yonsei Med. J..

[36] Nabe T. (2013). Tumor Necrosis Factor Alpha-Mediated Asthma?. Int. Arch. Allergy Immunol..

[37] Chen S., Saeed A.F.U.H., Liu Q., Jiang Q., Xu H., Xiao G.G., Rao L., Duo Y. (2023). Macrophages in Immunoregulation and Therapeutics. Signal Transduct. Target. Ther..

[38] Huang C., Wang J., Zheng X., Chen Y., Wei H., Sun R., Tian Z. (2018). Activation of TLR Signaling in Sensitization-Recruited Inflammatory Monocytes Attenuates OVA-Induced Allergic Asthma. Front. Immunol..

[39] Zuo L., Lucas K., Fortuna C.A., Chuang C.-C., Best T.M. (2015). Molecular Regulation of Toll-like Receptors in Asthma and COPD. Front. Physiol..

[40] Zakeri A., Russo M. (2018). Dual Role of Toll-like Receptors in Human and Experimental Asthma Models. Front. Immunol..

[41] Britt R.D., Thompson M.A., Sasse S., Pabelick C.M., Gerber A.N., Prakash Y.S. (2019). Th1 Cytokines TNF-α and IFN-γ Promote Corticosteroid Resistance in Developing Human Airway Smooth Muscle. Am. J. Physiol.-Lung Cell. Mol. Physiol..

[42] Ji T., Li H. (2023). T-Helper Cells and Their Cytokines in Pathogenesis and Treatment of Asthma. Front. Immunol..

[43] Deng Z., Ding W., Li F., Shen S., Huang C., Lai K. (2022). Pulmonary IFN-γ Causes Lymphocytic Inflammation and Cough Hypersensitivity by Increasing the Number of IFN-γ-Secreting T Lymphocytes. Allergy Asthma Immunol. Res..

[44] Makrinioti H., Bush A., Gern J., Johnston S.L., Papadopoulos N., Feleszko W., Camargo C.A., Hasegawa K., Jartti T. (2021). The Role of Interferons in Driving Susceptibility to Asthma Following Bronchiolitis: Controversies and Research Gaps. Front. Immunol..

[45] Yoshida M., Leigh R., Matsumoto K., Wattie J., Ellis R., O’Byrne P.M., Inman M.D. (2002). Effect of Interferon-γ on Allergic Airway Responses in Interferon-γ-Deficient Mice. Am. J. Respir. Crit. Care Med..

[46] Fahy J.V. (2015). Type 2 Inflammation in Asthma—Present in Most, Absent in Many. Nat. Rev. Immunol..

[47] Lloyd C.M., Hessel E.M. (2010). Functions of T Cells in Asthma: More than Just T_H_2 Cells. Nat. Rev. Immunol..

[48] Bradding P. (2007). Mast Cell Regulation of Airway Smooth Muscle Function in Asthma. Eur. Respir. J..

[49] Schroeder J.T. (2011). Basophils: Emerging Roles in the Pathogenesis of Allergic Disease. Immunol. Rev..

[50] Li Y.-F., Gauderman W.J., Avol E., Dubeau L., Gilliland F.D. (2006). Associations of Tumor Necrosis Factor G-308A with Childhood Asthma and Wheezing. Am. J. Respir. Crit. Care Med..

[51] Meulmeester F.L., Mailhot-Larouche S., Celis-Preciado C., Lemaire-Paquette S., Ramakrishnan S., Wechsler M.E., Brusselle G., Corren J., Hardy J., Diver S.E. (2025). Inflammatory and Clinical Risk Factors for Asthma Attacks (ORACLE2): A Patient-Level Meta-Analysis of Control Groups of 22 Randomised Trials. Lancet Respir. Med..

[52] Howell I., Howell A., Pavord I.D. (2023). Type 2 Inflammation and Biological Therapies in Asthma: Targeted Medicine Taking Flight. J. Exp. Med..

[53] Pelaia C., Pelaia G., Maglio A., Tinello C., Gallelli L., Lombardo N., Terracciano R., Vatrella A. (2023). Pathobiology of Type 2 Inflammation in Asthma and Nasal Polyposis. J. Clin. Med..

[54] Ricciardolo F.L.M., Sprio A.E., Baroso A., Gallo F., Riccardi E., Bertolini F., Carriero V., Arrigo E., Ciprandi G. (2021). Characterization of T2-Low and T2-High Asthma Phenotypes in Real-Life. Biomedicines.

[55] AlBloushi S., Al-Ahmad M. (2024). Exploring the Immunopathology of Type 2 Inflammatory Airway Diseases. Front. Immunol..

[56] Maison N., Omony J., Illi S., Thiele D., Skevaki C., Dittrich A.-M., Bahmer T., Rabe K.F., Weckmann M., Happle C. (2022). T2-High Asthma Phenotypes across Lifespan. Eur. Respir. J..

[57] Ogulur I., Mitamura Y., Yazici D., Pat Y., Ardicli S., Li M., D’Avino P., Beha C., Babayev H., Zhao B. (2025). Type 2 Immunity in Allergic Diseases. Cell. Mol. Immunol..

[58] Bakakos A., Anagnostopoulos N., Bakakos P. (2024). Eosinophils and T2 Inflammation in Severe Asthma. Explor. Asthma Allergy.

[59] Sim S., Choi Y., Park H.-S. (2024). Update on Inflammatory Biomarkers for Defining Asthma Phenotype. Allergy. Asthma Immunol. Res..

[60] Steinke J.W., Borish L. (2001). Th2 Cytokines and Asthma—Interleukin-4: Its Role in the Pathogenesis of Asthma, and Targeting It for Asthma Treatment with Interleukin-4 Receptor Antagonists. Respir. Res..

[61] Borish L.C., Nelson H.S., Lanz M.J., Claussen L., Whitmore J.B., Agosti J.M., Garrison L. (1999). Interleukin-4 Receptor in Moderate Atopic Asthma. A Phase I/II Randomized, Placebo-Controlled Trial. Am. J. Respir. Crit. Care Med..

[62] Pelaia C., Paoletti G., Puggioni F., Racca F., Pelaia G., Canonica G.W., Heffler E. (2019). Interleukin-5 in the Pathophysiology of Severe Asthma. Front. Physiol..

[63] Varricchi G., Bagnasco D., Borriello F., Heffler E., Canonica G.W. (2016). Interleukin-5 Pathway Inhibition in the Treatment of Eosinophilic Respiratory Disorders: Evidence and Unmet Needs. Curr. Opin. Allergy Clin. Immunol..

[64] Massey O.W., Suphioglu C. (2022). Taking a Breather: Advances in Interleukin 5 Inhibition for Asthma Relief. Int. J. Mol. Sci..

[65] Pelaia C., Heffler E., Crimi C., Maglio A., Vatrella A., Pelaia G., Canonica G.W. (2022). Interleukins 4 and 13 in Asthma: Key Pathophysiologic Cytokines and Druggable Molecular Targets. Front. Pharmacol..

[66] Kanoh S., Tanabe T., Rubin B.K. (2011). IL-13-induced MUC5AC Production and Goblet Cell Differentiation Is Steroid Resistant in Human Airway Cells. Clin. Exp. Allergy.

[67] Bergeron C., Tulic M.K., Hamid Q. (2010). Airway Remodelling in Asthma: From Benchside to Clinical Practice. Can. Respir. J..

[68] Chibana K., Trudeau J.B., Mustovich A.T., Hu H., Zhao J., Balzar S., Chu H.W., Wenzel S.E. (2008). IL-13 Induced Increases in Nitrite Levels Are Primarily Driven by Increases in Inducible Nitric Oxide Synthase as Compared with Effects on Arginases in Human Primary Bronchial Epithelial Cells. Clin. Exp. Allergy.

[69] Iwaszko M., Biały S., Bogunia-Kubik K. (2021). Significance of Interleukin (IL)-4 and IL-13 in Inflammatory Arthritis. Cells.

[70] Newcomb D.C., Peebles R.S. (2013). Th17-Mediated Inflammation in Asthma. Curr. Opin. Immunol..

[71] Al-Ramli W., Préfontaine D., Chouiali F., Martin J.G., Olivenstein R., Lemière C., Hamid Q. (2009). TH17-Associated Cytokines (IL-17A and IL-17F) in Severe Asthma. J. Allergy Clin. Immunol..

[72] Rahmawati S.F., Te Velde M., Kerstjens H.A.M., Dömling A.S.S., Groves M.R., Gosens R. (2021). Pharmacological Rationale for Targeting IL-17 in Asthma. Front. Allergy.

[73] Hough K.P., Curtiss M.L., Blain T.J., Liu R.-M., Trevor J., Deshane J.S., Thannickal V.J. (2020). Airway Remodeling in Asthma. Front. Med..

[74] Ramakrishnan R.K., Al Heialy S., Hamid Q. (2019). Role of IL-17 in Asthma Pathogenesis and Its Implications for the Clinic. Expert Rev. Respir. Med..

[75] Chesné J., Braza F., Mahay G., Brouard S., Aronica M., Magnan A. (2014). IL-17 in Severe Asthma. Where Do We Stand?. Am. J. Respir. Crit. Care Med..

[76] Gaffen S.L. (2009). Structure and Signalling in the IL-17 Receptor Family. Nat. Rev. Immunol..

[77] Syabbalo N. (2020). Mechanisms Of Interleukin -17 in the Pathogenesis of Neutrophilic Asthma. Pulm. Med. Respir. Res..

[78] Ricciardolo F.L.M., Sorbello V., Folino A., Gallo F., Massaglia G.M., Favatà G., Conticello S., Vallese D., Gani F., Malerba M. (2017). Identification of IL-17F/Frequent Exacerbator Endotype in Asthma. J. Allergy Clin. Immunol..

[79] Sorbello V., Ciprandi G., Di Stefano A., Massaglia G.M., Favatà G., Conticello S., Malerba M., Folkerts G., Profita M., Rolla G. (2015). Nasal IL-17F Is Related to Bronchial IL-17F/Neutrophilia and Exacerbations in Stable Atopic Severe Asthma. Allergy.

[80] Chenuet P., Fauconnier L., Madouri F., Marchiol T., Rouxel N., Ledru A., Mauny P., Lory R., Uttenhove C., van Snick J. (2017). Neutralization of Either IL-17A or IL-17F Is Sufficient to Inhibit House Dust Mite Induced Allergic Asthma in Mice. Clin. Sci..

[81] Manni M.L., Robinson K.M., Alcorn J.F. (2014). A Tale of Two Cytokines: IL-17 and IL-22 in Asthma and Infection. Expert Rev. Respir. Med..

[82] Margelidon-Cozzolino V., Tsicopoulos A., Chenivesse C., de Nadai P. (2022). Role of Th17 Cytokines in Airway Remodeling in Asthma and Therapy Perspectives. Front. Allergy.

[83] Dudakov J.A., Hanash A.M., van den Brink M.R.M. (2015). Interleukin-22: Immunobiology and Pathology. Annu. Rev. Immunol..

[84] Bafadhel M., McKenna S., Terry S., Mistry V., Pancholi M., Venge P., Lomas D.A., Barer M.R., Johnston S.L., Pavord I.D. (2012). Blood Eosinophils to Direct Corticosteroid Treatment of Exacerbations of Chronic Obstructive Pulmonary Disease. Am. J. Respir. Crit. Care Med..

[85] Ghosh S., Erzurum S.C. (2011). Nitric Oxide Metabolism in Asthma Pathophysiology. Biochim. Biophys. Acta (BBA) Gen. Subj..

[86] Ricciardolo F.L.M., Sorbello V., Ciprandi G. (2015). FeNO as Biomarker for Asthma Phenotyping and Management. Allergy Asthma Proc..

[87] Ahmad Al Obaidi A.H., Mohamed Al Samarai A.G., Yahya Al Samarai A.K., Al Janabi J.M. (2008). The Predictive Value of IgE as Biomarker in Asthma. J. Asthma.

[88] Izuhara K., Ohta S., Ono J. (2016). Using Periostin as a Biomarker in the Treatment of Asthma. Allergy. Asthma Immunol. Res..

[89] Sutton B.J., Davies A.M., Bax H.J., Karagiannis S.N. (2019). IgE Antibodies: From Structure to Function and Clinical Translation. Antibodies.

[90] Kuhl K., Hanania N.A. (2012). Targeting IgE in Asthma. Curr. Opin. Pulm. Med..

[91] Gill M.A., Bajwa G., George T.A., Dong C.C., Dougherty I.I., Jiang N., Gan V.N., Gruchalla R.S. (2010). Counterregulation between the FcεRI Pathway and Antiviral Responses in Human Plasmacytoid Dendritic Cells. J. Immunol..

[92] Gill M.A., Liu A.H., Calatroni A., Krouse R.Z., Shao B., Schiltz A., Gern J.E., Togias A., Busse W.W. (2018). Enhanced Plasmacytoid Dendritic Cell Antiviral Responses after Omalizumab. J. Allergy Clin. Immunol..

[93] Soler M., Matz J., Townley R., Buhl R., O’brien J., Fox H., Thirlwell J., Gupta N., Della Cioppa G.A. (2001). The Anti-IgE Antibody Omalizumab Reduces Exacerbations and Steroid Requirement in Allergic Asthmatics. Eur. Respir. J..

[94] Corren J., Casale T., Deniz Y., Ashby M. (2003). Omalizumab, a Recombinant Humanized Anti-IgE Antibody, Reduces Asthma-Related Emergency Room Visits and Hospitalizations in Patients with Allergic Asthma. J. Allergy Clin. Immunol..

[95] Humbert M., Beasley R., Ayres J., Slavin R., Hébert J., Bousquet J., Beeh K., Ramos S., Canonica G.W., Hedgecock S. (2005). Benefits of Omalizumab as Add-on Therapy in Patients with Severe Persistent Asthma Who Are Inadequately Controlled despite Best Available Therapy (GINA 2002 Step 4 Treatment): INNOVATE. Allergy.

[96] Normansell R., Walker S., Milan S.J., Walters E.H., Nair P. (2014). Omalizumab for Asthma in Adults and Children. Cochrane Database Syst. Rev..

[97] Braunstahl G.-J., Chen C.-W., Maykut R., Georgiou P., Peachey G., Bruce J. (2013). The EXpeRience Registry: The ‘Real-World’Effectiveness of Omalizumab in Allergic Asthma. Respir. Med..

[98] Djukanovic R., Wilson S.J., Kraft M., Jarjour N.N., Steel M., Chung K.F., Bao W., Fowler-Taylor A., Matthews J., Busse W.W. (2004). Effects of Treatment with Anti-Immunoglobulin E Antibody Omalizumab on Airway Inflammation in Allergic Asthma. Am. J. Respir. Crit. Care Med..

[99] Noga O., Hanf G., Brachmann I., Klucken A., Kleinetebbe J., Rosseau S., Kunkel G., Suttorp N., Seybold J. (2006). Effect of Omalizumab Treatment on Peripheral Eosinophil and T-Lymphocyte Function in Patients with Allergic Asthma. J. Allergy Clin. Immunol..

[100] Hoshino M., Ohtawa J. (2012). Effects of Adding Omalizumab, an Anti-Immunoglobulin E Antibody, on Airway Wall Thickening in Asthma. Respiration.

[101] Corren J., Casale T.B., Lanier B., Buhl R., Holgate S., Jimenez P. (2009). Safety and Tolerability of Omalizumab. Clin. Exp. Allergy.

[102] Gasser P., Tarchevskaya S.S., Guntern P., Brigger D., Ruppli R., Zbären N., Kleinboelting S., Heusser C., Jardetzky T.S., Eggel A. (2020). The Mechanistic and Functional Profile of the Therapeutic Anti-IgE Antibody Ligelizumab Differs from Omalizumab. Nat. Commun..

[103] Maurer M., Giménez-Arnau A.M., Sussman G., Metz M., Baker D.R., Bauer A., Bernstein J.A., Brehler R., Chu C.-Y., Chung W.-H. (2019). Ligelizumab for Chronic Spontaneous Urticaria. N. Engl. J. Med..

[104] Maurer M., Ensina L.F., Gimenez-Arnau A.M., Sussman G., Hide M., Saini S., Grattan C., Fomina D., Rigopoulos D., Berard F. (2024). Efficacy and Safety of Ligelizumab in Adults and Adolescents with Chronic Spontaneous Urticaria: Results of Two Phase 3 Randomised Controlled Trials. Lancet.

[105] Trischler J., Bottoli I., Janocha R., Heusser C., Jaumont X., Lowe P., Gautier A., Pethe A., Woessner R., Zerwes H.-G. (2021). Ligelizumab Treatment for Severe Asthma: Learnings from the Clinical Development Programme. Clin. Transl. Immunol..

[106] Wood R.A., Chinthrajah R.S., Eggel A., Bottoli I., Gautier A., Woisetschlaeger M., Tassinari P., Altman P. (2022). The Rationale for Development of Ligelizumab in Food Allergy. World Allergy Organ. J..

[107] Kuo B.-S., Li C.-H., Chen J.-B., Shiung Y.-Y., Chu C.-Y., Lee C.-H., Liu Y.-J., Kuo J.-H., Hsu C., Su H.-W. (2022). IgE-Neutralizing UB-221 MAb, Distinct from Omalizumab and Ligelizumab, Exhibits CD23-Mediated IgE Downregulation and Relieves Urticaria Symptoms. J. Clin. Investig..

[108] Walsh G.M. (2017). Biologics Targeting IL-5, IL-4 or IL-13 for the Treatment of Asthma—An Update. Expert Rev. Clin. Immunol..

[109] McCann M.R., Kosloski M.P., Xu C., Davis J.D., Kamal M.A. (2024). Dupilumab: Mechanism of Action, Clinical, and Translational Science. Clin. Transl. Sci..

[110] Castro M., Corren J., Pavord I.D., Maspero J., Wenzel S., Rabe K.F., Busse W.W., Ford L., Sher L., FitzGerald J.M. (2018). Dupilumab Efficacy and Safety in Moderate-to-Severe Uncontrolled Asthma. N. Engl. J. Med..

[111] Rabe K.F., Nair P., Brusselle G., Maspero J.F., Castro M., Sher L., Zhu H., Hamilton J.D., Swanson B.N., Khan A. (2018). Efficacy and Safety of Dupilumab in Glucocorticoid-Dependent Severe Asthma. N. Engl. J. Med..

[112] Wechsler M.E., Ford L.B., Maspero J.F., Pavord I.D., Papi A., Bourdin A., Watz H., Castro M., Nenasheva N.M., Tohda Y. (2022). Long-Term Safety and Efficacy of Dupilumab in Patients with Moderate-to-Severe Asthma (TRAVERSE): An Open-Label Extension Study. Lancet Respir. Med..

[113] Maspero J.F., Peters A.T., Chapman K.R., Domingo C., Stewart J., Hardin M., Maroni J., Tawo K., Khokhar F.A., Mortensen E. (2024). Long-Term Safety of Dupilumab in Patients With Moderate-to-Severe Asthma: TRAVERSE Continuation Study. J. Allergy Clin. Immunol. Pract..

[114] Wenzel S., Castro M., Corren J., Maspero J., Wang L., Zhang B., Pirozzi G., Sutherland E.R., Evans R.R., Joish V.N. (2016). Dupilumab Efficacy and Safety in Adults with Uncontrolled Persistent Asthma despite Use of Medium-to-High-Dose Inhaled Corticosteroids plus a Long-Acting Β2 Agonist: A Randomised Double-Blind Placebo-Controlled Pivotal Phase 2b Dose-Ranging Trial. Lancet.

[115] Corren J., Castro M., O’Riordan T., Hanania N.A., Pavord I.D., Quirce S., Chipps B.E., Wenzel S.E., Thangavelu K., Rice M.S. (2020). Dupilumab Efficacy in Patients with Uncontrolled, Moderate-to-Severe Allergic Asthma. J. Allergy Clin. Immunol. Pract..

[116] Busse W.W., Pavord I.D., Siddiqui S., Khan A.H., Praestgaard A., Nash S., Jacob-Nara J.A., Rowe P.J., Deniz Y. (2023). Dupilumab Improves Outcomes in Patients with Chronic Rhinosinusitis with Nasal Polyps and Coexisting Asthma Irrespective of Baseline Asthma Characteristics. J. Asthma Allergy.

[117] Pavord I.D., Casale T.B., Corren J., FitzGerald M.J., Deniz Y., Altincatal A., Gall R., Pandit-Abid N., Radwan A., Jacob-Nara J.A. (2024). Dupilumab Reduces Exacerbations Independent of Changes in Biomarkers in Moderate-to-Severe Asthma. J. Allergy Clin. Immunol. Pract..

[118] Hart T.K., Blackburn M.N., Brigham-Burke M., Dede K., Al-Mahdi N., Zia-Amirhosseini P., Cook R.M. (2002). Preclinical Efficacy and Safety of Pascolizumab (SB 240683): A Humanized Anti-Interleukin-4 Antibody with Therapeutic Potential in Asthma. Clin. Exp. Immunol..

[119] Paton N.I., Gurumurthy M., Lu Q., Leek F., Kwan P., Koh H.W.L., Molton J., Mortera L., Naval S., Bakar Z.A. (2024). Adjunctive Pascolizumab in Rifampicin-Susceptible Pulmonary Tuberculosis: Proof-of-Concept, Partially-Randomized, Double-Blind, Placebo-Controlled, Dose-Escalation Trial. J. Infect. Dis..

[120] (2001). A Phase I/II, Randomized, Double-Blind, Placebo-Controlled, Parallel-Group Pilot Study of SB 240683 in Patients with Symptomatic Steroid-Naive Asthma. https://clinicaltrials.gov/study/NCT00024544.

[121] Antoniu S.A. (2010). Pitrakinra, a Dual IL-4/IL-13 Antagonist for the Potential Treatment of Asthma and Eczema. Curr. Opin. Investig. Drugs.

[122] Wenzel S., Wilbraham D., Fuller R., Getz E.B., Longphre M. (2007). Effect of an Interleukin-4 Variant on Late Phase Asthmatic Response to Allergen Challenge in Asthmatic Patients: Results of Two Phase 2a Studies. Lancet.

[123] Otulana B.A., Wenzel S.E., Ind P.W., Bowden A., Puthukkeril S., Tomkinson A., Meyers D.A., Bleecker E.R., Yen Y.P. (2011). A Phase 2b Study of Inhaled Pitrakinra, An IL-4/IL-13 Antagonist, Successfully Identified Responder Subpopulations of Patients with Uncontrolled Asthma. D101. Asthma Genetics.

[124] Mukherjee M., Sehmi R., Nair P. (2014). Anti-IL5 Therapy for Asthma and Beyond. World Allergy Organ. J..

[125] Ortega H.G., Liu M.C., Pavord I.D., Brusselle G.G., FitzGerald J.M., Chetta A., Humbert M., Katz L.E., Keene O.N., Yancey S.W. (2014). Mepolizumab Treatment in Patients with Severe Eosinophilic Asthma. N. Engl. J. Med..

[126] Haldar P., Brightling C.E., Hargadon B., Gupta S., Monteiro W., Sousa A., Marshall R.P., Bradding P., Green R.H., Wardlaw A.J. (2009). Mepolizumab and Exacerbations of Refractory Eosinophilic Asthma. N. Engl. J. Med..

[127] Pavord I.D., Korn S., Howarth P., Bleecker E.R., Buhl R., Keene O.N., Ortega H., Chanez P. (2012). Mepolizumab for Severe Eosinophilic Asthma (DREAM): A Multicentre, Double-Blind, Placebo-Controlled Trial. Lancet.

[128] Ortega H.G., Yancey S.W., Mayer B., Gunsoy N.B., Keene O.N., Bleecker E.R., Brightling C.E., Pavord I.D. (2016). Severe Eosinophilic Asthma Treated with Mepolizumab Stratified by Baseline Eosinophil Thresholds: A Secondary Analysis of the DREAM and MENSA Studies. Lancet Respir. Med..

[129] Bel E.H., Wenzel S.E., Thompson P.J., Prazma C.M., Keene O.N., Yancey S.W., Ortega H.G., Pavord I.D. (2014). Oral Glucocorticoid-Sparing Effect of Mepolizumab in Eosinophilic Asthma. N. Engl. J. Med..

[130] Markham A. (2016). Reslizumab: First Global Approval. Drugs.

[131] Castro M., Zangrilli J., Wechsler M.E. (2015). Corrections. Reslizumab for Inadequately Controlled Asthma with Elevated Blood Eosinophil Counts: Results from Two Multicentre, Parallel, Double-Blind, Randomised, Placebo-Controlled, Phase 3 Trials. Lancet. Respir. Med..

[132] Corren J., Weinstein S., Janka L., Zangrilli J., Garin M. (2016). Phase 3 Study of Reslizumab in Patients With Poorly Controlled Asthma: Effects Across a Broad Range of Eosinophil Counts. Chest.

[133] Ibrahim H., O’Sullivan R., Casey D., Murphy J., MacSharry J., Plant B.J., Murphy D.M. (2019). The Effectiveness of Reslizumab in Severe Asthma Treatment: A Real-World Experience. Respir. Res..

[134] Hashimoto S., Kroes J.A., Eger K.A., Mau Asam P.F., Hofstee H.B., Bendien S.A., Braunstahl G.J., Broeders M.E.A.C., Imming L.M., Langeveld B. (2022). Real-World Effectiveness of Reslizumab in Patients With Severe Eosinophilic Asthma—First Initiators and Switchers. J. Allergy Clin. Immunol. Pract..

[135] Kolbeck R., Kozhich A., Koike M., Peng L., Andersson C.K., Damschroder M.M., Reed J.L., Woods R., Dall’Acqua W.W., Stephens G.L. (2010). MEDI-563, a Humanized Anti-IL-5 Receptor α MAb with Enhanced Antibody-Dependent Cell-Mediated Cytotoxicity Function. J. Allergy Clin. Immunol..

[136] Bleecker E.R., FitzGerald J.M., Chanez P., Papi A., Weinstein S.F., Barker P., Sproule S., Gilmartin G., Aurivillius M., Werkström V. (2016). Efficacy and Safety of Benralizumab for Patients with Severe Asthma Uncontrolled with High-Dosage Inhaled Corticosteroids and Long-Acting Β2-Agonists (SIROCCO): A Randomised, Multicentre, Placebo-Controlled Phase 3 Trial. Lancet.

[137] FitzGerald J.M., Bleecker E.R., Nair P., Korn S., Ohta K., Lommatzsch M., Ferguson G.T., Busse W.W., Barker P., Sproule S. (2016). Benralizumab, an Anti-Interleukin-5 Receptor α Monoclonal Antibody, as Add-on Treatment for Patients with Severe, Uncontrolled, Eosinophilic Asthma (CALIMA): A Randomised, Double-Blind, Placebo-Controlled Phase 3 Trial. Lancet.

[138] Busse W.W., Bleecker E.R., FitzGerald J.M., Ferguson G.T., Barker P., Sproule S., Olsson R.F., Martin U.J., Goldman M., Yañez A. (2019). Long-Term Safety and Efficacy of Benralizumab in Patients with Severe, Uncontrolled Asthma: 1-Year Results from the BORA Phase 3 Extension Trial. Lancet Respir. Med..

[139] FitzGerald J.M., Bleecker E.R., Menzies-Gow A., Zangrilli J.G., Hirsch I., Metcalfe P., Newbold P., Goldman M. (2018). Predictors of Enhanced Response with Benralizumab for Patients with Severe Asthma: Pooled Analysis of the SIROCCO and CALIMA Studies. Lancet Respir. Med..

[140] Nair P., Wenzel S., Rabe K.F., Bourdin A., Lugogo N.L., Kuna P., Barker P., Sproule S., Ponnarambil S., Goldman M. (2017). Oral Glucocorticoid–Sparing Effect of Benralizumab in Severe Asthma. N. Engl. J. Med..

[141] Ramakrishnan S., Russell R.E.K., Mahmood H.R., Krassowska K., Melhorn J., Mwasuku C., Pavord I.D., Bermejo-Sanchez L., Howell I., Mahdi M. (2025). Treating Eosinophilic Exacerbations of Asthma and COPD with Benralizumab (ABRA): A Double-Blind, Double-Dummy, Active Placebo-Controlled Randomised Trial. Lancet Respir. Med..

[142] Jackson D.J., Wechsler M.E., Jackson D.J., Bernstein D., Korn S., Pfeffer P.E., Chen R., Saito J., de Luíz Martinez G., Dymek L. (2024). Twice-Yearly Depemokimab in Severe Asthma with an Eosinophilic Phenotype. N. Engl. J. Med..

[143] Hershey G.K.K. (2003). IL-13 Receptors and Signaling Pathways: An Evolving Web. J. Allergy Clin. Immunol..

[144] Reza M.I., Kumar A., Pabelick C.M., Britt R.D., Prakash Y.S., Sathish V. (2024). Downregulation of Protein Phosphatase 2Aα in Asthmatic Airway Smooth Muscle. Am. J. Physiol.-Lung Cell. Mol. Physiol..

[145] Thomson N., Patel M., Smith A.D. (2012). Lebrikizumab in the Personalized Management of Asthma. Biol. Targets Ther..

[146] Corren J., Lemanske R.F., Hanania N.A., Korenblat P.E., Parsey M.V., Arron J.R., Harris J.M., Scheerens H., Wu L.C., Su Z. (2011). Lebrikizumab Treatment in Adults with Asthma. N. Engl. J. Med..

[147] Noonan M., Korenblat P., Mosesova S., Scheerens H., Arron J.R., Zheng Y., Putnam W.S., Parsey M.V., Bohen S.P., Matthews J.G. (2013). Dose-Ranging Study of Lebrikizumab in Asthmatic Patients Not Receiving Inhaled Steroids. J. Allergy Clin. Immunol..

[148] Hanania N.A., Noonan M., Corren J., Korenblat P., Zheng Y., Fischer S.K., Cheu M., Putnam W.S., Murray E., Scheerens H. (2015). Lebrikizumab in Moderate-to-Severe Asthma: Pooled Data from Two Randomised Placebo-Controlled Studies. Thorax.

[149] Scheerens H., Arron J.R., Zheng Y., Putnam W.S., Erickson R.W., Choy D.F., Harris J.M., Lee J., Jarjour N.N., Matthews J.G. (2014). The Effects of Lebrikizumab in Patients with Mild Asthma Following Whole Lung Allergen Challenge. Clin. Exp. Allergy.

[150] Piper E., Brightling C., Niven R., Oh C., Faggioni R., Poon K., She D., Kell C., May R.D., Geba G.P. (2013). A Phase II Placebo-Controlled Study of Tralokinumab in Moderate-to-Severe Asthma. Eur. Respir. J..

[151] Hanania N.A., Korenblat P., Chapman K.R., Bateman E.D., Kopecky P., Paggiaro P., Yokoyama A., Olsson J., Gray S., Holweg C.T.J. (2016). Efficacy and Safety of Lebrikizumab in Patients with Uncontrolled Asthma (LAVOLTA I and LAVOLTA II): Replicate, Phase 3, Randomised, Double-Blind, Placebo-Controlled Trials. Lancet Respir. Med..

[152] Corren J., Szefler S.J., Sher E., Korenblat P., Soong W., Hanania N.A., Berman G., Brusselle G., Zitnik R., Natalie C.R. (2024). Lebrikizumab in Uncontrolled Asthma: Reanalysis in a Well-Defined Type 2 Population. J. Allergy Clin. Immunol. Pract..

[153] Popovic B., Breed J., Rees D.G., Gardener M.J., Vinall L.M.K., Kemp B., Spooner J., Keen J., Minter R., Uddin F. (2017). Structural Characterisation Reveals Mechanism of IL-13-Neutralising Monoclonal Antibody Tralokinumab as Inhibition of Binding to IL-13Rα1 and IL-13Rα2. J. Mol. Biol..

[154] Brightling C.E., Chanez P., Leigh R., O’Byrne P.M., Korn S., She D., May R.D., Streicher K., Ranade K., Piper E. (2015). Efficacy and Safety of Tralokinumab in Patients with Severe Uncontrolled Asthma: A Randomised, Double-Blind, Placebo-Controlled, Phase 2b Trial. Lancet Respir. Med..

[155] Panettieri R.A., Sjöbring U., Péterffy A., Wessman P., Bowen K., Piper E., Colice G., Brightling C.E. (2018). Tralokinumab for Severe, Uncontrolled Asthma (STRATOS 1 and STRATOS 2): Two Randomised, Double-Blind, Placebo-Controlled, Phase 3 Clinical Trials. Lancet Respir. Med..

[156] Busse W.W., Brusselle G.G., Korn S., Kuna P., Magnan A., Cohen D., Bowen K., Piechowiak T., Wang M.M., Colice G. (2019). Tralokinumab Did Not Demonstrate Oral Corticosteroid-Sparing Effects in Severe Asthma. Eur. Respir. J..

[157] Corren J., Parnes J.R., Wang L., Mo M., Roseti S.L., Griffiths J.M., van der Merwe R. (2017). Tezepelumab in Adults with Uncontrolled Asthma. N. Engl. J. Med..

[158] Menzies-Gow A., Corren J., Bourdin A., Chupp G., Israel E., Wechsler M.E., Brightling C.E., Griffiths J.M., Hellqvist Å., Bowen K. (2021). Tezepelumab in Adults and Adolescents with Severe, Uncontrolled Asthma. N. Engl. J. Med..

[159] Corren J., Menzies-Gow A., Chupp G., Israel E., Korn S., Cook B., Ambrose C.S., Hellqvist Å., Roseti S.L., Molfino N.A. (2023). Efficacy of Tezepelumab in Severe, Uncontrolled Asthma: Pooled Analysis of the PATHWAY and NAVIGATOR Clinical Trials. Am. J. Respir. Crit. Care Med..

[160] Cox L.S. (2009). How Safe Are the Biologicals in Treating Asthma and Rhinitis?. Allergy Asthma Clin. Immunol..

[161] Akiho H. (2015). Promising Biological Therapies for Ulcerative Colitis: A Review of the Literature. World J. Gastrointest. Pathophysiol..

[162] Taillé C., Poulet C., Marchand-Adam S., Borie R., Dombret M.-C., Crestani B., Aubier M. (2013). Monoclonal Anti-TNF-α Antibodies for Severe Steroid-Dependent Asthma: A Case Series. Open Respir. Med. J..

[163] Cunningham G., Samaan M.A., Irving P.M. (2019). Golimumab in the Treatment of Ulcerative Colitis. Ther. Adv. Gastroenterol..

[164] Wenzel S.E., Barnes P.J., Bleecker E.R., Bousquet J., Busse W., Dahlén S.-E., Holgate S.T., Meyers D.A., Rabe K.F., Antczak A. (2009). A Randomized, Double-Blind, Placebo-Controlled Study of Tumor Necrosis Factor-α Blockade in Severe Persistent Asthma. Am. J. Respir. Crit. Care Med..

[165] Traczewski P., Rudnicka L. (2008). Adalimumab in Dermatology. Br. J. Clin. Pharmacol..

[166] Catal F., Mete E., Tayman C., Topal E., Albayrak A., Sert H. (2015). A Human Monoclonal Anti-TNF Alpha Antibody (Adalimumab) Reduces Airway Inflammation and Ameliorates Lung Histology in a Murine Model of Acute Asthma. Allergol. Immunopathol..

[167] Davis J.C., Van Der Heijde D., Braun J., Dougados M., Cush J., Clegg D.O., Kivitz A., Fleischmann R., Inman R., Tsuji W. (2003). Recombinant Human Tumor Necrosis Factor Receptor (Etanercept) for Treating Ankylosing Spondylitis: A Randomized, Controlled Trial. Arthritis Rheum..

[168] Holgate S.T., Noonan M., Chanez P., Busse W., Dupont L., Pavord I., Hakulinen A., Paolozzi L., Wajdula J., Zang C. (2011). Efficacy and Safety of Etanercept in Moderate-to-Severe Asthma: A Randomised, Controlled Trial. Eur. Respir. J..

[169] Borovcanin M.M., Minic Janicijevic S., Jovanovic I.P., Gajovic N.M., Jurisevic M.M., Arsenijevic N.N. (2020). Type 17 Immune Response Facilitates Progression of Inflammation and Correlates with Cognition in Stable Schizophrenia. Diagnostics.

[170] Hueber W., Patel D.D., Dryja T., Wright A.M., Koroleva I., Bruin G., Antoni C., Draelos Z., Gold M.H., Durez P. (2010). Effects of AIN457, a Fully Human Antibody to Interleukin-17A, on Psoriasis, Rheumatoid Arthritis, and Uveitis. Sci. Transl. Med..

[171] Vicovan A.G., Petrescu D.C., Cretu A., Ghiciuc C.M., Constantinescu D., Iftimi E., Strugariu G., Ancuta C.M., Caratașu C.-C., Solcan C. (2024). Targeting Common Inflammatory Mediators in Experimental Severe Asthma and Acute Lung Injury. Pharmaceuticals.

[172] Kirsten A., Watz H., Pedersen F., Holz O., Smith R., Bruin G., Koehne-Voss S., Magnussen H., Waltz D.A. (2013). The Anti-IL-17A Antibody Secukinumab Does Not Attenuate Ozone-Induced Airway Neutrophilia in Healthy Volunteers. Eur. Respir. J..

[173] Beck K.M., Koo J. (2019). Brodalumab for the Treatment of Plaque Psoriasis: Up-to-Date. Expert Opin. Biol. Ther..

[174] Busse W.W., Holgate S., Kerwin E., Chon Y., Feng J., Lin J., Lin S.-L. (2013). Randomized, Double-Blind, Placebo-Controlled Study of Brodalumab, a Human Anti–IL-17 Receptor Monoclonal Antibody, in Moderate to Severe Asthma. Am. J. Respir. Crit. Care Med..

[175] Toussirot E. (2018). Ixekizumab: An Anti- IL-17A Monoclonal Antibody for the Treatment of Psoriatic Arthritis. Expert Opin. Biol. Ther..

[176] Genovese M.C., Mysler E., Tomita T., Papp K.A., Salvarani C., Schwartzman S., Gallo G., Patel H., Lisse J.R., Kronbergs A. (2020). Safety of Ixekizumab in Adult Patients with Plaque Psoriasis, Psoriatic Arthritis and Axial Spondyloarthritis: Data from 21 Clinical Trials. Rheumatology.

[177] Griffiths C.E.M., Gooderham M., Colombel J.-F., Terui T., Accioly A.P., Gallo G., Zhu D., Blauvelt A. (2022). Safety of Ixekizumab in Adult Patients with Moderate-to-Severe Psoriasis: Data from 17 Clinical Trials with Over 18,000 Patient-Years of Exposure. Dermatol. Ther..

[178] Saint-Pierre M.D. (2022). Concomitant Use of Mepolizumab and Ixekizumab in a Patient with Severe Eosinophilic Asthma and Psoriasis: A Case Study. A65. Pulmonary Clinical Cases II.

[179] Adamič N., Vengust M. (2023). Regenerative Medicine in Lung Diseases: A Systematic Review. Front. Vet. Sci..

[180] Hussen B.M., Najmadden Z.B., Abdullah S.R., Rasul M.F., Mustafa S.A., Ghafouri-Fard S., Taheri M. (2024). CRISPR/Cas9 Gene Editing: A Novel Strategy for Fighting Drug Resistance in Respiratory Disorders. Cell Commun. Signal..

[181] Cereta A.D., Oliveira V.R., Costa I.P., Afonso J.P.R., Fonseca A.L., de Souza A.R.T., Silva G.A.M., Mello D.A.C.P.G., de Oliveira L.V.F., da Palma R.K. (2021). Emerging Cell-Based Therapies in Chronic Lung Diseases: What About Asthma?. Front. Pharmacol..

[182] Mishra B., Singh J. (2020). Novel Drug Delivery Systems and Significance in Respiratory Diseases. Targeting Chronic Inflammatory Lung Diseases Using Advanced Drug Delivery Systems.

[183] da Silva A.L., Silva L.A., Cruz F.F., Rocco P.R.M., Morales M.M. (2020). Application of Novel Nanotechnologies in Asthma. Ann. Transl. Med..

[184] Gysens F., Mestdagh P., de Bony de Lavergne E., Maes T. (2022). Unlocking the Secrets of Long Non-Coding RNAs in Asthma. Thorax.

